# Altered expression of long noncoding RNAs regulating neutrophilic inflammation in peripheral blood was associated with symptom severity in patients with house dust mite-induced allergic rhinitis

**DOI:** 10.3389/falgy.2024.1466480

**Published:** 2024-10-25

**Authors:** Jinming Zhao, Xiaoyu Pu, Xiangdong Wang, Luo Zhang

**Affiliations:** ^1^ Department of Otolaryngology Head and Neck Surgery, Beijing Tongren Hospital, Capital Medical University, Beijing, China; ^2^ Beijing Laboratory of Allergic Diseases, Beijing Municipal Education Commission and Beijing Key Laboratory of Nasal Diseases, Beijing Institute of Otolaryngology, Beijing, China; ^3^ Department of Allergy, Beijing Tongren Hospital, Capital Medical University, Beijing, China; ^4^ Research Unit of Diagnosis and Treatment of Chronic Nasal Diseases, Chinese Academy of Medical Sciences, Beijing, China

**Keywords:** allergic rhinitis, house dust mite, lncRNA, RNA-seq, peripheral leukocyte

## Abstract

**Background:**

Long noncoding RNAs (lncRNAs) have been implicated in a diverse array of human immune diseases; however, a comprehensive understanding of the expression and function of lncRNAs in the peripheral blood leukocytes of individuals suffering from house dust mite (HDM)-induced allergic rhinitis (AR) remains elusive.

**Objective:**

To explore the potential roles and functions of long noncoding RNAs (lncRNAs) in the pathogenesis of AR.

**Methods:**

Sequencing analysis was performed on peripheral blood leukocytes collected from patients with HDM-induced AR and healthy controls (HCs) to elucidate the expression patterns of lncRNAs. Differentially expressed (DE) lncRNAs were identified and validated, and further correlation analyses were conducted to explore their associations with visual analog scale (VAS) scores and cytokine levels in the serum and nasal secretions. Additionally, bioinformatics analyses were performed to predict the potential pathways influenced by DE lncRNAs. Finally, the diagnostic potential of these lncRNAs in AR was assessed via receiver operating characteristic (ROC) curve analysis.

**Results:**

Significant differences in the expression profiles of lncRNAs and mRNAs were detected between AR patients and HCs. Four lncRNAs were markedly upregulated in AR patients. AC011524.2 was positively correlated with nasal pruritus (*r* = 0.4492, *P* = 0.0411). AL133371.3 was positively correlated with runny nose (*r* = 0.4889, *P* = 0.0245). AC011524.2 was positively correlated with CXCL8 (*r* = 0.4504, *P* = 0.0035). AL133371.3 was significantly positively correlated with only IL-17 (*r* = 0.4028, *P* = 0.0100). IL-4 in the serum was positively related to IL-17 in the serum (*r* = 0.4163, *P* = 0.0002). CXCL5 in the serum was positively correlated with IFN-γ (*r* = 0.3336, *P* = 0.0354) in nasal secretions. The area under the curve (AUC) of the ROC curve resulting from the integration of the 4 lncRNAs exhibited a remarkable value of 0.940 for AR diagnosis.

**Conclusions:**

Our results identified several lncRNAs associated with AR symptoms and inflammatory cytokines. Specifically, AC011524.2 and AL133371.3 exhibited strong correlations with diverse AR manifestations and serum cytokines, suggesting their pivotal role in the pathogenesis of AR, likely via neutrophil- and Th17-related pathways. However, the precise underlying mechanisms are still elusive, necessitating further exploration.

## Introduction

Allergic rhinitis (AR), a prevalent condition, frequently coexists with chronic rhinosinusitis, asthma, conjunctivitis and other diseases (1). In China, the self-reported incidence of AR has increased from 11.1% in 2005 to 17.6% in 2011, significantly affecting people's quality of life (2). The etiology of AR, akin to other allergic conditions, encompasses intricate and multifactorial elements (3). AR can be categorized as persistent or seasonal, with house dust mites (HDMs) being the most common allergen (4). In particular, more than 90% of AR cases in central and southern China are attributed to HDMs (5). These allergens are ubiquitous and present throughout the year. Therefore, patients with HDM-induced AR have persistent allergic inflammation (6). Consequently, further research on HDM-induced AR is imperative to enhance our comprehension of its pathogenesis and improve strategies.

Long noncoding RNAs (lncRNAs) exceeding 200 nucleotides in length, occupy an important position in cellular regulation (7). They are integral to modulating gene expression, chromatin remodeling, cell cycle progression, and numerous other essential biological functions (8). Recent research has underscored the significant involvement of lncRNAs in various pathological processes, including malignancies, cardiovascular diseases, and neurodegenerative disorders (9). Their crucial regulatory role extends to the immune response, inflammatory processes and allergic reactions. For example, the lncRNA ANRIL is positively correlated with nasal symptom severity and the expression of inflammatory cytokines such as TNF-α, IL-4, IL-6, IL-13, and IL-17, while inversely correlated with the levels of anti-inflammatory cytokines like IL-10 and IFN-γ in the nasal mucosal cells of AR patients and healthy controls (HCs) (10). Yue et al. found that LNC-000632 expression was downregulated in nasal mucosal samples from AR patients and in IL-13-stimulated nasal epithelial cells (11). The lncRNA GATA3-AS1 and the GATA3 gene are regulated by the same transcriptional regulatory element, which may play important roles in the Th2 immune response (12). These findings towards the significance of differentially expressed (DE) lncRNAs in the pathogenesis of AR. Further exploration of lncRNA expression and function in AR, as well as elucidation of the underlying mechanism, could contribute to novel therapeutic strategies. The investigation of the interplay between lncRNAs and HDM-induced AR is still in its infancy (13).

Obtaining nasal mucosa samples poses clinical challenges, and for AR, specific IgE testing remains the primary diagnostic tool. Although peripheral blood from AR patients is readily accessible, the relationship between lncRNAs in AR peripheral blood leukocytes and various serum inflammatory cytokines remains unexplored. Therefore, investigating AR peripheral blood lncRNAs, their regulation of cytokines, and their clinical correlations is imperative. This study aimed to elucidate the role of lncRNAs in AR by contrasting their expression in peripheral blood leukocytes from AR patients with that in HCs. Furthermore, we determined the correlations among DE lncRNAs, clinical manifestations, and serum inflammatory factors. This research holds significant value, as identifying lncRNAs associated with AR symptoms could deepen our understanding of the molecular basis of this disease, ultimately contributing to enhanced diagnostic and therapeutic strategies.

## Methods

### Patient population

**Figure 1:**
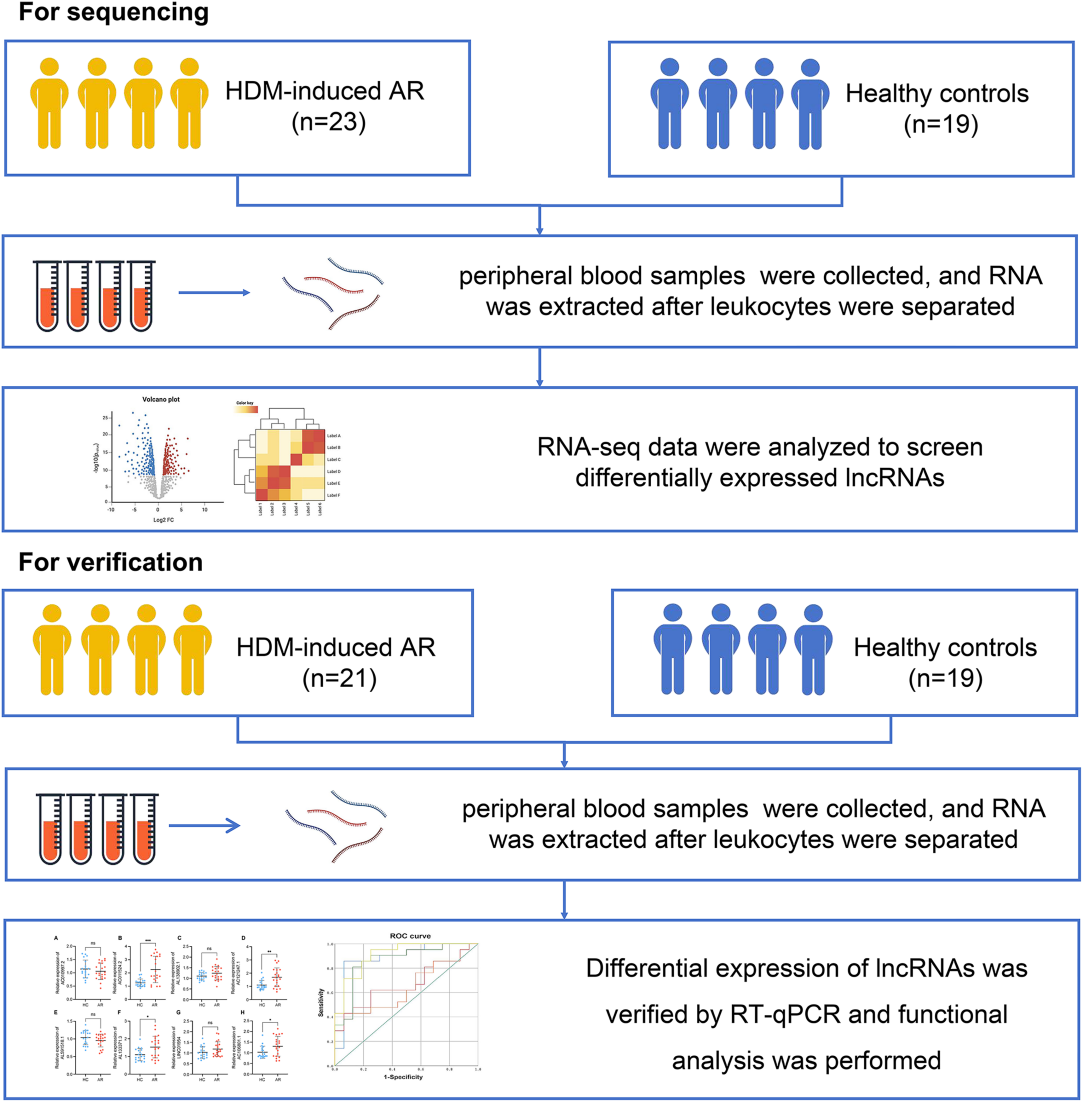
Overview of the research process.

A total of 23 HDM-induced AR patients and 19 HCs were included in this study. All patients were diagnosed with AR in the Department of Otolaryngology Head and Neck Surgery, Beijing Tongren Hospital, Capital Medical University, from May 2018 to May 2019. For verification, information on 21 patients with HDM-induced AR and 19 HCs were collected under the same conditions from September 2020 to October 2021 (Figure 1).

### Inclusion and exclusion criteria

The following inclusion criteria were used for HDM-induced AR: (1) met the diagnostic criteria for persistent AR (14); (2) patients with AR were primarily allergic to Dermatophagoides pteronyssinus (D1), Dermatophagoides farina (D2) or both, and the symptoms were caused by HDM; the serum specific immunoglobulin E (IgE) to D1 or D2 was equal to or greater than grade 2, as measured with ImmunoCAP Phadiatop (Thermo Fisher Scientific) (14, 15); (3) aged between 18 and 60 years, regardless of sex; and (4) did not use anti-allergic or steroid medications within the past 3 months. The following exclusion criteria were used: (1) patients who had undergone specific immunotherapy in the past; (2) patients with chronic rhinosinusitis, asthma, etc.; (3) patients with other immune-related diseases, tumors, chronic infectious diseases, hematologic disorders, etc.; and (4) pregnant or lactating women.

The following inclusion criteria were used for HCs: (1) absence of AR symptoms or history; (2) aged between 18 and 60 years, regardless of sex; and (3) not having used antiallergic or steroid medications within the past 3 months. The following exclusion criteria were used for HCs: (1) had chronic rhinosinusitis, asthma, etc.; (2) had other immune-related diseases, tumors, chronic infectious diseases, hematologic disorders, etc.; and (3) were pregnant or lactating.

### Visual analog scale (VAS)

The VAS was used to assess the overall severity of symptoms during allergic rhinitis episodes in the past week and included 12 assessment items: nasal congestion, nasal itchiness, sneezing, runny nose, itchy eyes, tearing, red eyes, eye pain, cough, breath holding, wheezing, and pressure sensation. The VAS score ranged from 0 (absence of symptoms) to 10 cm (very severe symptoms) for all combined nasal symptoms. All the subjects completed the VAS score at enrollment. The more severe the symptoms, the higher the score, with a maximum score of 120.

### Methods of nasal secretion collection

Nasal secretions were collected from AR patients and HCs via a polyvinyl alcohol medical sponge (Medtronic, 400402) into each patient's left and right nasal total nasal passages and left in place for 5–10 min to be removed when the sponge adsorbed nasal secretions until fully expanded. The sponges were subsequently placed into a centrifuge tube, which was weighed before and after collection to calculate the secretion content. One milliliter of saline was added, and the mixture was refrigerated at 4°C for 2 hours and centrifuged at 1500 × g for 15 min. The supernatant was retained and stored at −80°C.

### Serum-specific IgE detection

Blood samples were collected in 5-ml vacuum tubes and centrifuged to separate the serum. An ImmunoCAP Phadiatop (Thermo Fisher Scientific) was used to detect allergen-specific IgE antibodies in human serum, including for HDM (D1 and D2), cat dander, dog dander, *Quercus alba*, *Ulmus americana*, *Platanus acerifolia*, *Salix caprea*, *Populus deltoides*, mountain juniper, common silver birch, mugwort, Japanese Hop, ragweed, *Penicillium chrysogenum, Cladosporium herbarum*, *Aspergillus fumigatus* and *Alternaria alternata*. The instrument used was Phadia information and data management software, and all calculations were performed automatically. The qualitative results were positive or negative, and the clinical threshold was 0.35 kU/L with 6 grades (grade 0: 0.00–0.34 kUA/L; grade 1: 0.35–0.69 kUA/L; grade 2: 0.70–3.49 kUA/L; grade 3: 3.50–17.49 kUA/L; grade 4: 17.5–49.9 kUA/L; grade 5: 50.0–100.0 kUA/L; and grade 6: >100.0 kUA/L) (14,15).

### Generation of sequencing libraries from total RNA

Blood samples were collected in 5-ml EDTA vacuum tubes, and the leukocytes were separated via red blood cell lysis solution (Solarbio, R1010). Total RNA was extracted from leukocytes with Tri®-Reagent (Sigma, T9424) within 2 h. All procedures were performed according to the manufacturer's instructions. An RNA-seq library was constructed and sequenced by Novogene Company Limited. The NEBNext® UltraTM RNA Library Prep Kit (New England Biolabs) was used for library construction. After the library was constructed, an Agilent 2100 Bioanalyzer (Agilent Technologies) was used to determine the size of the library insert. Reverse transcription‒quantitative polymerase chain reaction (RT‒qPCR) was used to quantify the effective concentration of the library accurately to ensure quality. After qualified library inspection, Illumina sequencing was performed for the different pooled libraries according to the requirements of the effective concentration and target onboard data amount, and a 150 bp matching end reading was produced. The basic principle of sequencing is sequencing by synthesis.

### Analysis of DE genes

Differential expression analysis was performed between two comparisons via the DESeq2 R package (1.16.1). DESeq2 provides statistical procedures to determine differential expression in digital gene expression data using a model based on a negative binomial distribution. The method of Benjamini and Hochberg was used to adjust the *P* value to control the error detection rate. Genes with adjusted *P* < 0.05 were considered DE according to DESeq2. For each sequenced library, the read count was adjusted via a scale normalization factor in the edgeR package prior to differential expression analysis. Differential expression analysis of the two conditions was performed via the edgeR software package. The Benjamini and Hochberg methods were used to adjust the *P* values. After correction, the *P* value and fold change were used as thresholds for significant differential expression.

### Functional enrichment analyses

The Kyoto Encyclopedia of Genes and Genomes (KEGG) and Gene Ontology (GO) databases were used for functional enrichment analysis of the DE lncRNAs. The significance of the biological pathways is indicated by the *P* value. The *P* value was corrected via the Benjamin and Hochberg multiple tests, and the Q value was obtained.

### RT-qPCR validation

The quality of the total RNA was assessed via a Nanodrop 2000 (Thermo Fisher Scientific), and complementary DNA was synthesized from 1 μg of total RNA via PrimeScript RT Master Mix (Takara, RR036A). RT‒qPCR was performed via SYBR Green mix (ABclonal Biotechnology, RK21203) to determine gene expression levels. The reaction was performed using a StepOnePlus^TM^ Real-Time PCR System (Applied Biosystems). The average transcript levels of the genes were normalized to those of GAPDH. The primers used are listed in Supplementary Table S1.

### Measurement of cytokine expression in serum and nasal secretions

We used the Human Cytokine A Premixed Magnetic Luminex® Performance Assay (R&D Systems, FCSTM03) to quantitatively detect a variety of cytokines and chemokines in serum and nasal secretions. Before Luminex testing, the sample was slowly thawed and pretreated according to the manufacturer's instructions. Each sample and standard were double-replicated in a 96-well plate. The concentration of each target protein in the serum samples was calculated via a standard curve. The results are expressed in pg/ml and were used for subsequent statistical analysis. The factors detected included C-X-C motif chemokine ligand 5 (CXCL5)/epithelial neutrophil-activating protein 78 (ENA-78), basic fibroblast growth factor (FGF basic), granulocyte colony stimulating factor (G-CSF), granulocyte‒macrophage colony stimulating factor (GM-CSF), interferon-γ (IFN-γ), interleukin (IL)-1β/IL-1F2, IL-2, IL-4, IL-5, IL-6, CXCL8/IL-8, IL-10, IL-17, CC chemokine ligand 2 (CCL2)/monocyte chemoattractant protein-1 (MCP-1), CCL3/macrophage inflammatory protein (MIP-1ɑ), CCL4/MIP-1β, CCL5/RANTES, tumor necrosis factor-ɑ (TNF-ɑ), thrombopoietin (Tpo), and vascular endothelial cell growth factor (VEGF).

### Statistical analysis

SPSS 16.0 for Windows was used for receiver operating characteristic (ROC) analyses, and other statistical analyses were performed via GraphPad Prism 7.0 software. R studio software was used to construct heatmaps and volcano plots. For normally distributed data, the mean ± standard deviation (SD) was used. Correlational analyses were performed via Spearman rank correlation analysis. Comparisons between the 2 groups were made via the independent samples t test or Mann‒Whitney *U* test. In all the cases, a *P* value < 0.05 was considered statistically significant.

## Results

### Characteristics of the study population

A total of 19 HCs and 23 AR patients were included in the first stage of this study. All AR patients were diagnosed in the Department of Otolaryngology, Beijing Tongren Hospital, Capital Medical University, from May 2018 to May 2019. The patients were diagnosed with HDM-induced AR via the UniCAP system. The total IgE, D1 and D2 values of the AR group were all positive (Table 1). There were no significant differences in age or sex distribution between the AR patients and HCs (*P* > 0.05). The serum D1, D2 and total IgE values in the AR group were significantly different from those in the HC group (*P* < 0.05).

**Table 1:** Demographic data of HDM-induced AR patients and HCs.

Characteristic	For sequencing		For verification		*P* value			
	AR (*n* = 23)	HC (*n* = 19)	AR (*n* = 21)	HC (*n* = 19)	a	b	c	d
Sex (male/female)	14/9	16/3	10/11	13/6	0.1485	0.2164	0.5452	0.4470
Age (y)	33.83 ± 14.12	34.00 ± 13.04	38.19 ± 12.96	42.05 ± 13.22	0.9481	0.3571	0.2931	0.0783
Smoking history (Yes/No)	6/17	5/14	4/17	7/12	0.2585	0.2933	0.7240	0.7281
Drinking history (Yes/No)	8/15	3/16	4/17	2/17	0.2905	0.6642	0.3182	0.6928
HDM only^a^ (Yes/No)	5/18	–	3/19	–	–	–	0.6995	–
D1 (kUA/L)	13.02 ± 17.94	0.02 ± 0.02	6.57 ± 7.69	0.04 ± 0.05	**0**.**0012**	**0**.**0007**	0.0727	0.1364
D2 (kUA/L)	12.58 ± 11.39	0.04 ± 0.03	7.36 ± 9.70	0.04 ± 0.04	**<0.0001**	**0**.**0027**	0.1106	0.8057
Total IgE (kUA/L)	406.75 ± 96.04	19.40 ± 6.75	311.09 ± 246.61	25.12 ± 13.42	**<0.0001**	**<0**.**0001**	0.0920	0.1055

The bold text indicates significance. The data are expressed as the means ± standard deviations (SDs). “a” represents the *P* value for the comparison between the AR and HC in sequencing; “b” represents the *P* value for the comparison between the AR and HC in verification; “c” represents the *P* value for the comparison between the AR of sequencing and AR of verification; ‘d” represents the *P* value for the comparison between the HC of sequencing and HC of verification. HDM, house dust mite; AR, allergic rhinitis; HC, healthy control; IgE, immunoglobulin E; D1, Dermatophagoides pteronyssinus; D2, Dermatophagoides farina.

^a^ HDM only means positive serum specific IgE of D1 and/or D2.

### The expression profiles of lncRNAs and mRNAs in the peripheral blood leukocytes of AR patients and HCs differed

**Figure 2:**
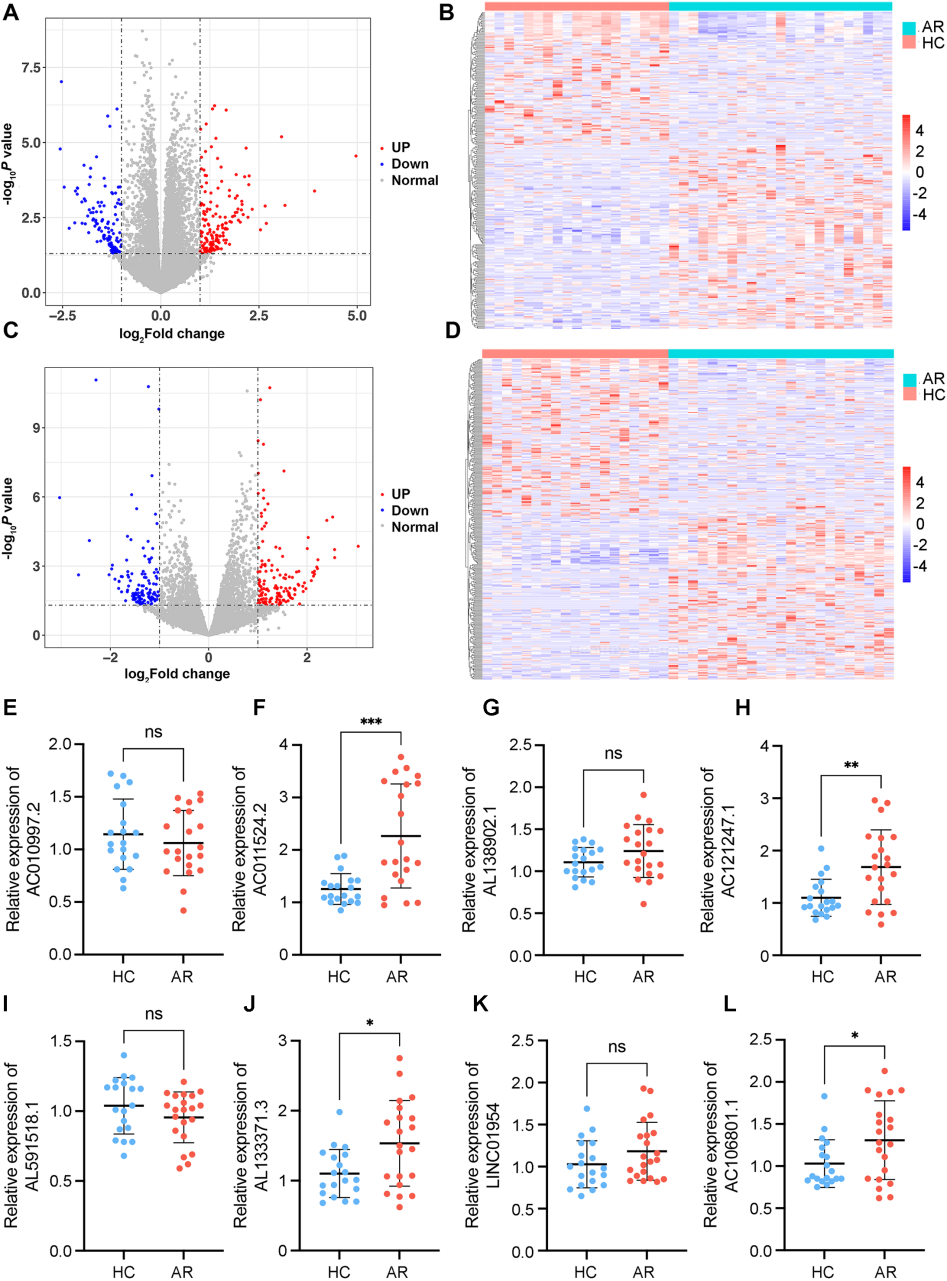
Identification of DE mRNAs and lncRNAs. **(A)** Volcano plot of DE mRNAs. **(B)** Heatmaps of DE mRNAs between the AR and healthy control groups (|log_2_ fold change | >1 and *P* < 0.05). **(C)** Volcano plot of DE lncRNAs. **(D)** Heatmaps of DE lncRNAs between the AR and healthy control groups (|log_2_ fold change| >1 and *P* < 0.05). In each heatmap, AR indicates the HDM-induced AR group, and HC indicates the healthy control group. In the volcano map, the red dots represent upregulated genes, and the blue dots represent downregulated genes. **(E**–**L)** Relative expression levels of the top 8 DE lncRNAs determined via RT–qPCR. AR indicates the HDM-induced AR group, and HC indicates the healthy control group. *P < 0.05, **P < 0.01, ***P < 0.001.

To investigate differences in the peripheral blood leukocyte lncRNA expression profiles between the HDM-induced AR group and the HC group, each RNA sample extracted from peripheral blood leukocytes was sequenced via the Illumina HiSeq platform, and the data were calculated as feature counts. The expression levels of lncRNAs and mRNAs in each sample were calculated, and DE lncRNAs were identified via multiple differential comparisons. There were obvious differences between the 350 lncRNAs (|log_2_ fold change| >1, *P* < 0.05), including 170 downregulated and 180 upregulated lncRNAs (Supplementary Table S2) (Figures 2A,B). There were obvious differences between 298 mRNAs (|log_2_ fold change| >1, *P* < 0.05), including 125 downregulated and 173 upregulated mRNAs (|log_2_ fold change| >1, *P* < 0.05) **(**Supplementary Table S3) (Figures 2C,D**)**.

### Validation of DE lncRNAs

On the basis of the results of differential gene analysis, the 8 most significantly upregulated lncRNAs were selected as validation targets for further experimental confirmation. A total of 21 AR patients and 19 HCs were further included according to the same inclusion and exclusion criteria (Table 1). The 8 most significantly upregulated lncRNAs were detected via RT‒qPCR: AC010997.2, AC011524.2, AL138902.1, AC121247.1, AL591518.1, AL133371.3, LINC01954, and AC106801.1. AC011524.2, AL138902.1, AC121247.1, AL133371.3, LINC01954, and AC106801.1 were upregulated in the peripheral blood leukocytes of AR patients compared with those of HCs (Figures 2E–L). The results were consistent with the RNA-seq results, with AC011524.2, AC121247.1, AL133371.3, and AC106801.1 showing significant differences (*P* < 0.05) (Figures 2F,H,J,L).

### Correlation analysis between serum IgE levels, DE lncRNAs, and clinical symptoms

To understand the associations between DE lncRNAs, serum IgE levels, and clinical symptoms and to evaluate whether these lncRNAs are involved in AR and may influence patient symptom presentation, we conducted a correlation analysis. The correlation analysis results revealed that AC011524.2 was positively correlated with nasal pruritus (*r* = 0.4492, *P* = 0.0411) and itchy eyes (*r* = 0.4515, *P* = 0.0399). There was no significant correlation between AC121247.1 and symptom scores (*P* > 0.05). AL133371.3 was positively correlated with runny nose (*r* = 0.4889, *P* = 0.0245) but not significantly correlated with other symptoms (*P* > 0.05). AC106801.1 was positively correlated with nasal pruritus (*r* = 0.4682, *P* = 0.0323), sneezing (*r* = 0.4776, *P* = 0.0286), and itching (*r* = 0.4709, *P* = 0.0312) but was not significantly correlated with other symptoms (Table 2). However, no significant correlation was found between the 4 DE lncRNAs and total serum IgE, D1 or D2 (Supplementary Table S4). Similarly, correlation analysis between the VAS score and total IgE, D1, and D2 also revealed no significant correlations (Supplementary Table S5).

**Table 2:** Correlation analysis between VAS score and differentially expressed lncRNAs.

VAS score	AR	AC011524.2		AC121247.1		AL133371.3		AC106801.1	
		r	*P*	r	*P*	r	*P*	r	*P*
Total score	32.05 ± 6.27	0.2928	0.3317	−0.0337	0.8846	0.3330	0.1402	0.3470	0.1233
Nasal obstruction	6.60 ± 1.70	−0.0411	0.8594	0.0897	0.6990	−0.1316	0.5696	0.0796	0.7314
Nasal pruritus	3.86 ± 0.71	0.4492	**0**.**0411**	−0.1803	0.4342	−0.2327	0.3101	0.4682	**0**.**0323**
Sneeze	5.71 ± 2.58	0.3234	0.1527	−0.2878	0.2059	0.0573	0.8051	0.4776	**0**.**0286**
Runny nose	5.23 ± 2.99	−0.1141	0.6223	−0.0122	0.9578	0.4889	**0**.**0245**	0.1308	0.5721
Itchy eyes	4.31 ± 2.01	0.4515	**0**.**0399**	−0.2320	0.3116	−0.0807	0.7280	0.4709	**0**.**0312**
Tearing	3.71 ± 2.05	0.1179	0.6107	0.3056	0.1779	0.3253	0.1502	−0.3497	0.1202
Red eyes	1.33 ± 1.63	−0.3564	0.1128	−0.0141	0.9515	0.2018	0.3803	−0.1133	0.6248
Eye pain	0.48 ± 0.70	−0.3711	0.0976	0.1743	0.4498	−0.0232	0.9205	−0.1136	0.6240
Cough	0.38 ± 0.74	0.0245	0.9161	0.2324	0.3106	0.3010	0.1850	0.1241	0.5920
Breath holding	0.22 ± 0.48	−0.0279	0.9043	0.08170	0.7248	−0.1652	0.4742	−0.1278	0.5810
Wheezing	0.13 ± 0.48	−0.0097	0.9669	−0.0052	0.9821	−0.1158	0.6172	0.1403	0.5441
Pressure sensation	0.17 ± 0.46	−0.2280	0.3202	−0.2094	0.3623	−0.0075	0.9744	−0.1117	0.6299

The bold text indicates significance. The data are expressed as the means ± standard deviations (SDs).

### Correlation analysis of DE lncRNAs and inflammatory cytokines in the serum

**Figure 3:**
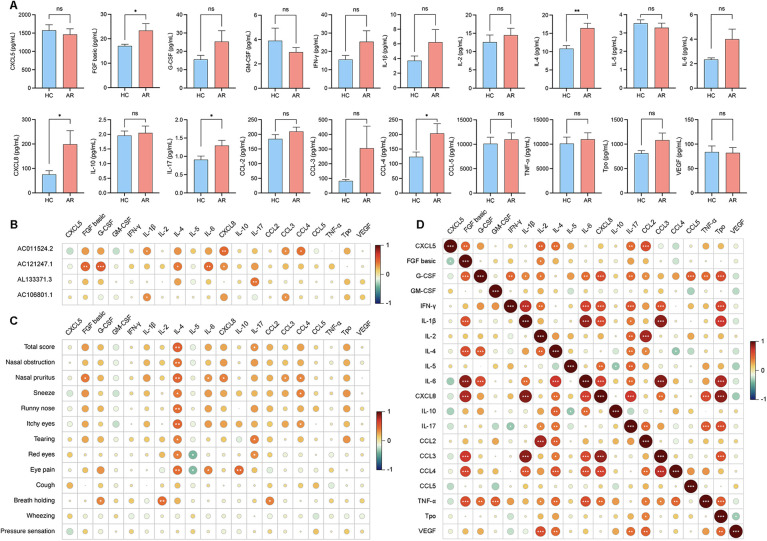
**(A)** Levels of cytokine expression in serum. **(B)** Correlation analysis of the 4 DE lncRNAs with serum inflammatory cytokines. **(C)** Correlation analysis between the VAS score and the levels of inflammatory cytokines in the serum. **(D)** Correlation analysis between each cytokine in the serum.

To investigate the potential involvement of lncRNAs in AR and understand their associations with serum inflammatory cytokines, we conducted an analysis. The analysis of serum inflammatory cytokine levels revealed that the levels of basic FGF, IL-4, CXCL8, IL-17 and CCL3 were significantly greater in the AR group than in the HC group, whereas the other cytokines did not show significant differences (Figure 3A; Supplementary Table S6). The expression of AC011524.2 was positively correlated with that of IL-1β (*r* = 0.3362, *P* = 0.0349), CXCL8 (*r* = 0.4504, *P* = 0.0035), CCL3 (*r* = 0.3344, *P* = 0.0349), and CCL4 (*r* = 0.4010, *P* = 0.0103). AC121247.1 was significantly positively correlated with FGF basic (*r* = 0.4697, *P* = 0.0022), G-CSF (*r* = 0.5130, *P* = 0.0007), IL-4 (*r* = 0.3903, *P* = 0.0128), IL-6 (*r* = 0.4415, *P* = 0.0044), and CXCL8 (r = 0.3210, *P* = 0.0434). AL133371.3 was significantly positively correlated with only IL-17 (*r* = 0.4028, *P* = 0.0100). AC106801.1 was significantly positively correlated with IL-1β (*r* = 0.3274, *P* = 0.0392) and CCL3 (*r* = 0.3138, *P* = 0.0487) (Figure 3B; Table 3).

**Table 3:** Correlation analysis of serum inflammatory cytokines and differential expression of lncRNA.

Inflammatory cytokine	AC011524.2		AC121247.1		AL133371.3		AC106801.1	
	r	*P*	r	*P*	r	*P*	r	*P*
CXCL5	−0.2368	0.1412	−0.1271	0.4345	−0.0572	0.7261	0.0558	0.7325
FGF basic	0.3000	0.0600	0.4697	**0**.**0022**	0.1946	0.2288	0.1194	0.4630
G-CSF	0.2687	0.0937	0.5130	**0**.**0007**	0.1711	0.2913	−0.1383	0.3946
GM-CSF	−0.2334	0.1472	−0.0884	0.5876	−0.0140	0.9319	−0.1152	0.4792
IFN-γ	0.0926	0.5698	0.1242	0.4452	−0.0651	0.6896	−0.0167	0.9187
IL-1β	0.3362	**0**.**0339**	0.1680	0.3000	−0.0183	0.9109	0.3274	**0**.**0392**
IL-2	−0.0238	0.8839	0.1213	0.4560	−0.0045	0.9779	−0.0267	0.8700
IL-4	0.3007	0.0593	0.3903	**0**.**0128**	0.2664	0.0966	0.0305	0.8517
IL-5	0.1451	0.3718	−0.1054	0.5173	−0.1343	0.4087	−0.0243	0.8817
IL-6	0.2783	0.0821	0.4415	**0**.**0044**	0.1464	0.3673	0.0641	0.6945
CXCL8	0.4504	**0**.**0035**	0.3210	**0**.**0434**	0.0062	0.9696	0.2937	0.0658
IL-10	0.0722	0.6579	0.1055	0.5172	0.0747	0.6470	−0.0589	0.7182
IL-17	0.1638	0.3125	0.2863	0.0732	0.4028	**0**.**0100**	−0.1592	0.3264
CCL2	0.0465	0.7756	0.0684	0.6751	0.0854	0.6005	0.0323	0.8429
CCL3	0.3344	**0**.**0349**	0.1947	0.2287	−0.0589	0.7183	0.3138	**0**.**0487**
CCL4	0.4010	**0**.**0103**	0.1677	0.3010	0.0799	0.6239	0.0671	0.6809
CCL5	−0.1257	0.4396	0.0890	0.5850	−0.0191	0.9069	−0.0470	0.7734
TNF-α	−0.0006	0.9970	0.1742	0.2824	−0.0321	0.8441	−0.0351	0.8296
Tpo	0.2342	0.1458	0.0038	0.9816	0.1906	0.2387	0.1841	0.2554
VEGF	0.0128	0.9375	−0.0328	0.8407	0.1329	0.4136	0.1430	0.3788

The bold text indicates significance. “a” represents the r and *P* value for the comparison between cytokine concentration and relative expression of AC011524.2; “b” represents the r and *P* value for the comparison between cytokine concentration and relative expression of AC121247.1; “c” represents the r and *P* value for the comparison between cytokine concentration and relative expression of AL133371.3; “d” represents the r and *P* value for the comparison between cytokine concentration and relative expression of AC106801.1.

### Correlation analysis of inflammatory cytokines in the serum with clinical symptoms

To investigate the associations between inflammatory cytokines in serum and nasal secretions and clinical symptoms and to understand how these cytokines influence symptom manifestation in AR patients, we conducted a correlation analysis. IL-4 in the serum was positively correlated with the total score (*r* = 0.4912, *P* = 0.0013) and nasal pruritus (*r* = 0.4731, *P* = 0.0020), IL-17 in the serum was positively correlated with the total score (*r* = 0.3127, *P* = 0.0045), CCL3 in the serum was positively correlated with nasal pruritus (*r* = 0.3141, *P* = 0.0484), and CCL4 in the serum was positively correlated with nasal pruritus (*r* = 0.3596, *P* = 0.0227) (Figure 3C; Supplementary Table S7).

### Correlation analysis between inflammatory factors in the serum

To explore the interactions between inflammatory factors in the serum and to understand how they collectively influence immune and inflammatory responses in AR patients, we conducted a correlation analysis. FGF basic was positively correlated with CXCL8 (*r* = 0.7850, *P* < 0.0001), IL-4 was positively correlated with IL-17 (*r* = 0.4163, *P* = 0.0002), CXCL8 was positively correlated with IL-1β (*r* = 0.8885, *P* < 0.0001), and CCL3 was positively correlated with CXCL8 (*r* = 0.8580, *P* < 0.0001) (Figure 3D; Supplementary Table S8).

### Correlation analysis of inflammatory cytokines in nasal secretions and serum

**Figure 4:**
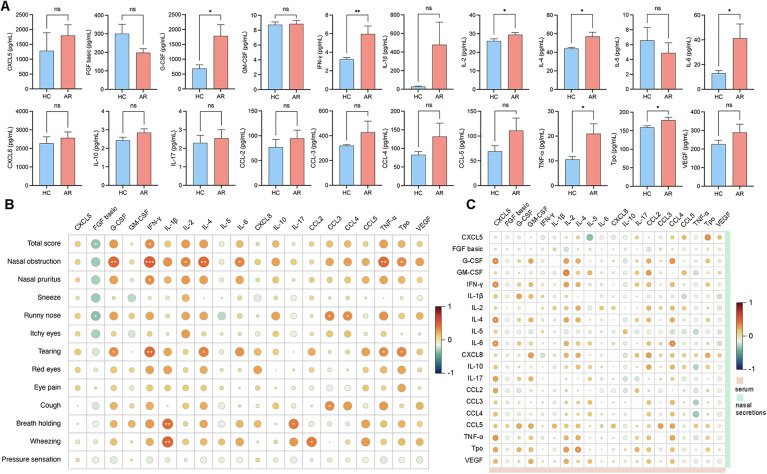
**(A)** Levels of cytokine expression in nasal secretions. **(B)** Correlation analysis between the VAS score and the levels of inflammatory cytokines in nasal secretions. **(C)** Correlation analysis between serum cytokines and nasal secretion cytokines.

To explore the associations between inflammatory factors in nasal secretions and serum and to understand the interactions and underlying mechanisms of these factors in AR patients, we performed a correlation analysis. The analysis results revealed that G-CSF, IFN-γ, IL-4, IL-6 and TNF-ɑ were significantly elevated in patients with AR nasal secretions (Figure 4A; Supplementary Table S9). IFN-γ in nasal secretions was positively correlated with nasal obstruction (*r* = 0.5025, *P* = 0.0010) and nasal pruritus (*r* = 0.3264, *P* = 0.0398). IL-4 in nasal secretions was positively correlated with nasal obstruction (*r* = 0.4492, *P* = 0.0036). IL-6 in nasal secretions was positively correlated with nasal obstruction (*r* = 0.3516, *P* = 0.0261). TNF-α was positively correlated with nasal obstruction (*r* = 0.4389, *P* = 0.0046) (Figure 4B; Supplementary Table S10). CXCL5 in the serum was positively correlated with G-CSF (*r* = 0.3274, *P* = 0.0392), IFN-γ (*r* = 0.3336, *P* = 0.0354), IL-4 (*r* = 0.3209, *P* = 0.0435), and IL-6 (*r* = 0.3182, *P* = 0.0454) in nasal secretions. Serum IL-4 was positively correlated with Tpo (*r* = 0.3242, *P* = 0.0416) in nasal secretions. Serum IL-5 was negatively correlated with CXCL5 (*r* = −0.3372, *P* = 0.0164) in nasal secretions. Serum CCL4 was positively correlated with IL-6 (*r* = 0.3342, *P* = 0.0351) in nasal secretions. Serum TNF-ɑ was negatively correlated with CCL4 (*r* = −0.3350, *P* = 0.0346) in nasal secretions. Serum Tpo was positively correlated with CXCL5 (*r* = 0.3327, *P* = 0.0360) in nasal secretions (Figure 4C; Supplementary Table S11).

### Biological pathway analysis of significantly DE lncRNAs

**Figure 5:**
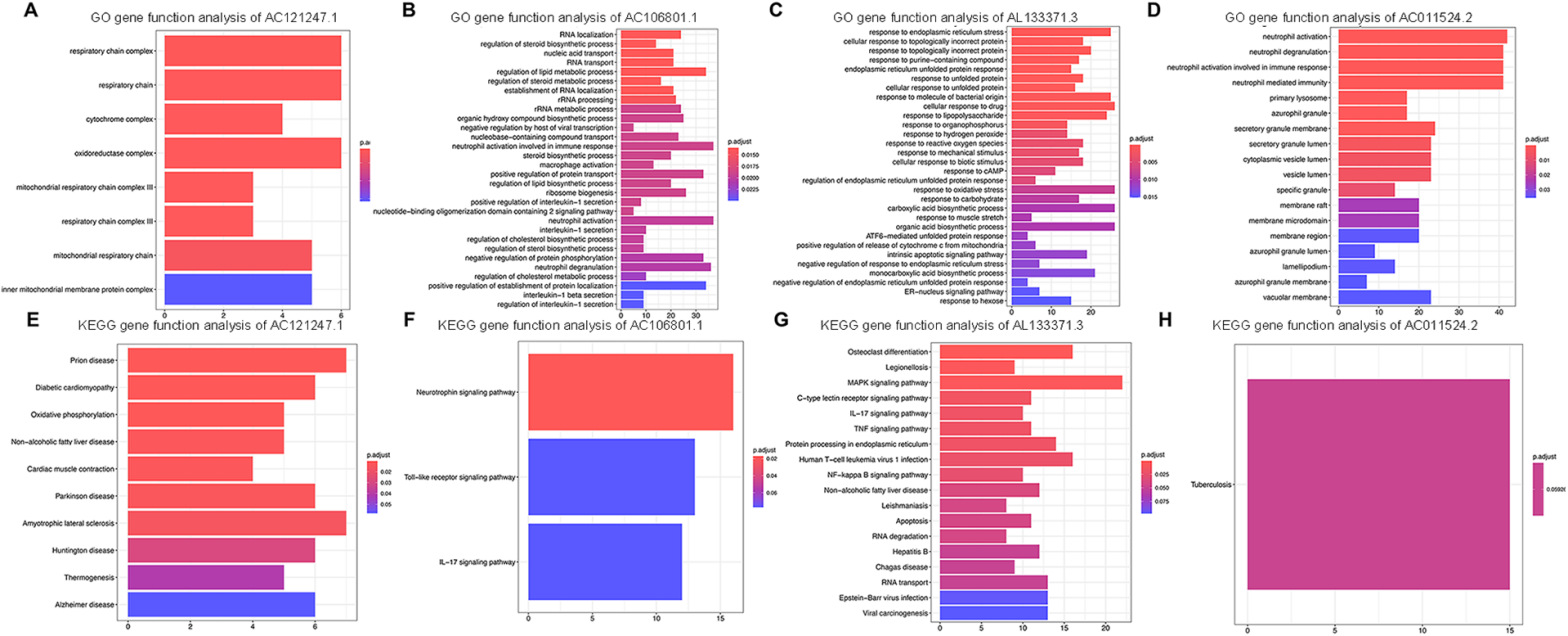
Go and KEGG analyses of DE lncRNAs. **(A)** GO gene function analysis of AC121247.1. **(B)** GO gene function analysis of AC106801.1. **(C)** GO gene function analysis of AL133371.3. **(D)** GO gene function analysis of AC011524.2. **(E)** KEGG gene function analysis of AC121247.1. **(F)** KEGG gene function analysis of AC106801.1. **(G)** KEGG gene function analysis of AL133371.3. **(H)** KEGG gene function analysis of AC011524.2.

To investigate the potential metabolic pathways and biological processes in which these lncRNAs may be involved, the potential functions of the lncRNAs were predicted via the GO and KEGG pathway annotations of their coexpressed mRNAs via the DAVID online tool, and possible biological pathways were identified. GO and KEGG analyses revealed that the DE genes were significantly enriched in multiple pathways involved in the immune response, such as the Toll-like receptor signaling pathway, the IL-17 signaling pathway and neutrophil activation. Overall, AC121247.1, AC106801.1, AL133371.3, and AC011524.2 may participate in the immune response (Figure 5).

### Predictive properties of lncRNA levels in the diagnosis of AR

**Figure 6:**
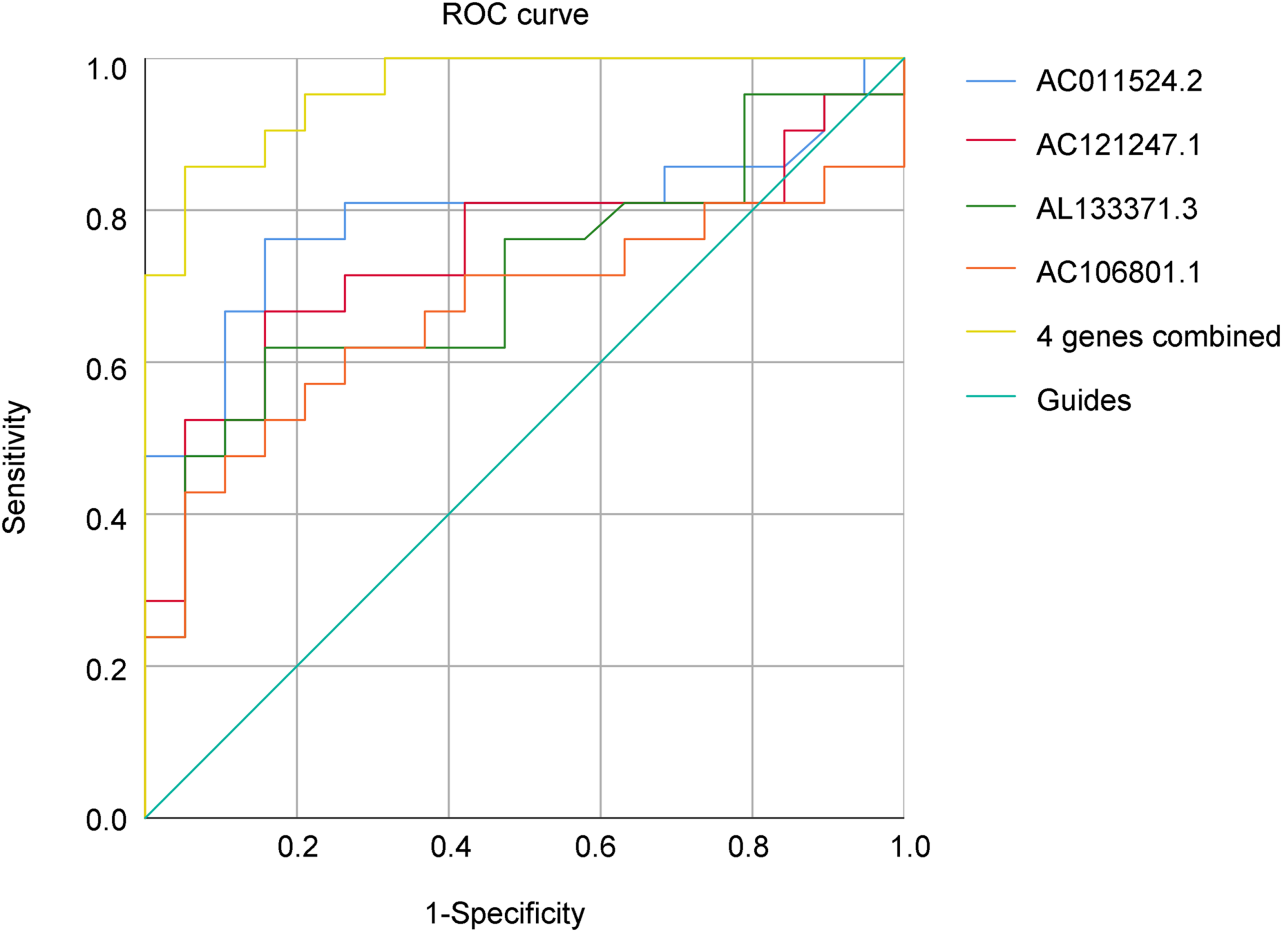
ROC curve of DE lncRNAs.

The area under the curve (AUC) of a single lncRNA was between 0.667 and 0.788; the highest AUC was 0.788 for AC011524.2, and the sensitivity and specificity were 0.762 and 0.842, respectively. When the 4 genes were combined, the AUC was significantly increased to 0.940, indicating greater sensitivity and specificity, and the Youden index reached 0.714, indicating that this combination method had greater accuracy in identifying AR (Figure 6; Table 4).

**Table 4:** Sensitivity and specificity of differentially expressed lncRNAs.

Gene ID	AUC	Sensitivity	Specificity	Youden index
AC011524.2	0.788	0.762	0.842	0.604
AC121247.1	0.702	0.619	0.842	0.509
AL133371.3	0.677	0.619	0.789	0.408
AC106801.1	0.647	0.429	0.894	0.323
4 genes combined	0.940	0.810	0.947	0.714

AUC, area under the curve.

## Discussion

The positive findings of specific IgE in serum offer a clear indication of the primary allergens, while their levels serve as an objective gauge of the body's sensitization status. However, in clinical practice, the level of a specific IgE does not always align with the severity of the condition, and positive results do not necessarily align with the gravity of clinical symptoms (14–17). Our study concurs with this, finding no significant correlation. However, our research has uncovered numerous DE lncRNAs that exhibit a connection with AR symptoms.

Our study revealed that AC011524.2 was positively correlated with nasal pruritus and smell dysfunction. Additionally, AC011524.2 was positively correlated with CXCL8 and CCL3. Meanwhile, CXCL8 was positively correlated with nasal pruritus. Moreover, AC011524.2 was enriched in pathways related to neutrophil activation, neutrophil degranulation, and other immune response pathways. These findings indicate that AC011524.2 might be a pivotal factor in the pathogenesis of AR by influencing neutrophil-related processes. Historically, neutrophils have been considered relatively understated white blood cells within the intricate immune system (18). However, an increasing number of studies have identified their significant involvement in allergic respiratory diseases (19–24), especially in AR (24–26). Following continuous, low-dose exposure to HDM allergens, neutrophils in the nasal mucosa undergo notable elevation, culminating in a peak count during the heightened state of HDM allergic inflammation, which suggests a correlation between the level of neutrophils and the severity of AR (25, 26). CXCL8 and CCL3 are both neutrophil-associated factors (27), and CXCL8 significantly increases in various bodily fluids after sensitization, such as sputum (28, 29), nasal secretions (30), and serum (31), which may be related to neutrophil chemotaxis during allergic reactions. AC011524.2 may affect the activation and degranulation process of neutrophils in peripheral blood by regulating the expression of these cytokines, thus contributing to the occurrence and development of AR. While we observed a correlation between AC011524.2 and various clinical symptoms and inflammatory factors, the specific regulatory mechanism involved remains elusive. Our previous studies revealed that neutrophils can express matrix metalloproteinases (MMPs), such as MMP-8, in nasal polyps (32), which indicates a similar remodeling process by neutrophils in AR combined with the above findings in the present study. Therefore, future research should focus on the intricate molecular mechanisms governing how AC011524.2 modulates neutrophils and their related pathways, ultimately fostering the emergence and progression of AR.

Our findings indicated that AL133371.3 was positively correlated with runny nose. Additionally, AL133371.3 was significantly positively correlated with only IL-17. Furthermore, AL133371.3 was enriched in the IL-17 signaling pathway in the present study, suggesting that it may be involved in the expression of IL-17 and the development of AR. The discovery of Th17 and regulatory T cells has provided a valuable addition to the established Th1/Th2 imbalance theory, especially as Th17 cells are believed to be linked to chronic allergic reactions (33–35). IL-17 actively contributes to inflammatory responses, specifically by promoting the recruitment and activation of neutrophils and triggering the generation of significant amounts of IL-6, CXCL8 and granulocyte colony-stimulating factor (34, 36). Many studies have shown that IL-17 is associated with the severity of AR symptoms (37–39). It has also been shown that the Th17 cytokines IL-17 and TGF-β1 promote the expression of Th2-related factors (40). Therefore, AL133371.3 may be involved in the development of AR by affecting the expression of IL-17.

Concurrently, we observed significant increases in the serum IL-4, CXCL8/IL-8, and IL-17 levels in AR patients. IL-4, a pivotal cytokine in type 2 inflammatory responses, plays a role in the formation of polyclonal IgE, facilitating the class switch of B cells toward IgE production (41). CXCL8/IL-8 and IL-17 are key cytokines involved in the type 3 inflammatory response, which can promote neutrophil recruitment and participate in angiogenesis. These key cytokines can promote chemotactic eosinophils and T lymphocytes, thereby promoting the occurrence and development of AR (31, 33–35). In addition, the level of the chemokine CCL3, a key regulator of the immune microenvironment, is significantly elevated in the serum of AR patients (42), which has also been confirmed in animal experiments (43). CCL3 synthesis is important for maintaining the recruitment of inflammatory cells during episodes of inflammation, as well as for activating eosinophils and T cells and regulating Ig production. CCL3 constitutes a second signal for the degranulation of mast cells, acting as a direct costimulatory signal via CCR1, making this chemokine crucial for inducing acute-phase AR (42–44).

By correlation analysis of serum inflammatory factors, IL-4 and IL-17 were found to be positively correlated in the serum of AR patients. The interaction between the Th2 and Th17 pathways in patients with AR is very complex. Sun et al. demonstrated in a mouse model that Th17 cytokines positively affect the differentiation of CD4+ cells and promote the production of IL-4 (45), which aggravates the symptoms associated with AR, a result that is consistent with the results of the present study. However, Choy et al. reported an inhibitory effect of IL-4 and IL-13 on the Th17 response in a mouse model of asthma (46), which is consistent with our previous findings in patients with CRSwNP (40), indicating that the Th2 and Th17 pathways are mutually exclusive and regulate each other.

Meanwhile, we found that G-CSF, IFN-γ, IL-4, IL-6 and TNF-ɑ were significantly increased in AR nasal secretions, suggesting the occurrence of local inflammatory response in the nasal cavity (47). In addition, all of these significantly elevated factors were positively correlated with the symptoms of nasal obstruction in AR, which may be due to the stimulation of HDM to the monocyte-macrophage in the nasal mucosa to produce G-CSF, IFN-γ and TNF-ɑ, and increased secretion of IL-4, IL-6 by upregulating B lymphocytes, mast cells and basophils, and induced VCAM-1 on vascular endothelium, thus directing inflammatory cells to migrate to the site of inflammation and causing exacerbation of nasal symptoms (48, 49).

Research on lncRNAs in AR has been widely reported. Ma et al. investigated the changes in the expression of lncRNAs in nasal mucosal samples from 4 HCs and 4 patients with AR. The study revealed a total of 2,259 lncRNAs detected in these samples, and GO and pathway analyses revealed enrichment in several biological processes and cellular signaling pathways related to AR development, such as positive regulation of IL-13 secretion, the Fc epsilon RI signaling pathway and the NF-kappa B signaling pathway (50). Peripheral blood mononuclear cells (PBMCs) from 3 AR patients and 3 HCs were collected and analyzed via microarray detection and verified in 16 AR patients and 18 HCs. They demonstrated that 31 lncRNAs were DE in the PBMCs of patients with AR and that 4 DE lncRNAs may be related to inflammation and the immune response (51). While prior investigations delved into the significance of lncRNAs in AR, they focused primarily on PBMCs, potentially neglecting the crucial role played by granulocytes, notably neutrophils. Our decision to focus on peripheral blood leukocytes offers a broader perspective on the immune and inflammatory profiles of AR patients. This study marks the first instance of uncovering the potential of lncRNAs in blood to modulate the severity of AR through neutrophils, thereby augmenting the knowledge base of previous research.

This study has notable limitations. First, it was conducted with a restricted sample size confined to a single center, excluding patients from diverse geographical locations. Consequently, the small sample size hindered the ability to further categorize patients into subgroups. Additionally, the study duration was prolonged due to the COVID-19 pandemic, which hindered its continuation for an extended period. Furthermore, the study lacked an in-depth exploration of the role of lncRNAs, and the intricate mechanism between DE lncRNAs and AR remains to be elucidated.

Our study revealed novel upregulated lncRNAs in the peripheral blood leukocytes of AR patients, highlighting their potential significance in AR, particularly via neutrophil-related processes and the IL-17 signaling pathway. Notably, AC011524.2 and AL133371.3 displayed remarkable correlations with AR symptoms and inflammatory cytokines, indicating their involvement in AR severity. In addition, many neutrophil-related cytokines are significantly elevated in the serum, and these cytokines are associated with various clinical symptoms of AR. These findings suggest that cytokines in serum may be influenced by the expression of specific lncRNAs. Despite the inevitable limitations of our study, these findings significantly contribute to the current understanding of lncRNAs in AR and lay the groundwork for future endeavors to decipher the intricate mechanisms and therapeutic implications of these lncRNAs in AR.

## Data Availability

The datasets presented in this study can be found in online repositories. The names of the repository/repositories and accession number(s) can be found in the article/Supplementary Material.
