# Supplementary material for: Altered expression of long noncoding RNAs regulating neutrophilic inflammation in peripheral blood was associated with symptom severity in patients with house dust mite-induced allergic rhinitis
Source: Front Allergy. 2024 Oct 25;5:1466480. doi: 10.3389/falgy.2024.1466480 (PMC11543571; doi:10.3389/falgy.2024.1466480)
Supplement: Supplementary file 1 [file Table1.docx]

Supplementary Material

**Table S1** Sequences of the primers used for RT‒qPCR validation.

| ENSG ID | Gene symbol | Forward primer（5’-3’） | Reverse primer（5’-3’） |
| --- | --- | --- | --- |
| ENSG00000236842 | AC010997.2 | TTAACTGGCATCGTCTCT | GCTTGTCATCTTCCACTCT |
| ENSG00000267240 | AC011524.2 | GGAAGCAAGATGGAGTCA | ACAGGGCTACCAGAAATG |
| ENSG00000232298 | AL138902.1 | GCGGAAGGACATCTGTAT | GCGGAAGGACATCTGTAT |
| ENSG00000225399 | AC121247.1 | TTCCAAGCCAGTACAGTAA | AACTCCACCATAATGAGACTT |
| ENSG00000284999 | AL591518.1 | GTGTTACCGAACCATTAGAAT | CAACCAAGACATAGAAGAATCA |
| ENSG00000259130 | AL133371.3 | CAGCAGAATGGTGAGCAAG | GGGAAGGAATGAAAGTAATAGTAT |
| ENSG00000271952 | LINC01954 | GATATGAGACAGCCAGATTG | AGTCACGCAACTTGTAAG |
| ENSG00000253519 | AC106801.1 | ATCCAGCCTCTAGTCTCT | TGTAGGAATAGTCAGCATAGT |

**Table S2** LncRNAs differentially expressed between healthy controls and AR.

| ENSG ID | Gene symbol | log_2-_fold change | *P* |
| --- | --- | --- | --- |
| ENSG00000130600 | H19 | 1.4185 | **0.0184** |
| ENSG00000132204 | LINC00470 | 1.0883 | **0.0114** |
| ENSG00000177596 | AL355390.1 | 1.0883 | **0.0348** |
| ENSG00000203496 | AL133216.1 | 1.5309 | **0.0098** |
| ENSG00000203620 | AL354919.1 | 1.2667 | **0.0428** |
| ENSG00000205054 | LINC01121 | 1.3463 | **0.0285** |
| ENSG00000205628 | LINC01446 | 1.4858 | **0.0462** |
| ENSG00000220161 | LINC02076 | 1.2419 | **0.0317** |
| ENSG00000223813 | AC007255.1 | 1.2523 | **0.0428** |
| ENSG00000223823 | LINC01342 | 1.0860 | **0.0029** |
| ENSG00000223882 | ABCC5-AS1 | 1.4488 | **0.0178** |
| ENSG00000223911 | AC009480.1 | 1.4765 | **0.0087** |
| ENSG00000224079 | AC091729.1 | 1.6330 | **0.0073** |
| ENSG00000224116 | INHBA-AS1 | 1.0920 | **0.0226** |
| ENSG00000224292 | AF196972.1 | 1.1311 | **0.0089** |
| ENSG00000224513 | AC109309.1 | 1.8300 | **0.0148** |
| ENSG00000224958 | PGM5-AS1 | 1.2185 | **0.0463** |
| ENSG00000225399 | AC121247.1 | 2.2223 | **0.0004** |
| ENSG00000225807 | AC069281.1 | 2.1482 | **0.0005** |
| ENSG00000226476 | LINC01748 | 1.9119 | **0.0031** |
| ENSG00000226496 | LINC00323 | 1.2685 | **0.0303** |
| ENSG00000226779 | NAALADL2-AS2 | 1.4519 | **0.0274** |
| ENSG00000226957 | AL358075.1 | 1.9491 | **0.0022** |
| ENSG00000227188 | MGAT3-AS1 | 1.2368 | **0.0428** |
| ENSG00000227589 | AL136528.1 | 1.6147 | **0.0101** |
| ENSG00000227619 | AL391056.1 | 1.3551 | **0.0399** |
| ENSG00000227676 | LINC01068 | 1.2592 | **0.0455** |
| ENSG00000228352 | AL354989.1 | 1.2620 | **0.0369** |
| ENSG00000228437 | LINC02474 | 1.1696 | **0.0456** |
| ENSG00000228549 | BX284668.2 | 1.3459 | **0.0121** |
| ENSG00000229086 | LINC01548 | 1.6589 | **0.0101** |
| ENSG00000229246 | LINC00377 | 1.3748 | **0.0236** |
| ENSG00000229953 | AL590666.2 | 1.7407 | **0.0036** |
| ENSG00000230174 | LINC01149 | 1.2859 | **0.0260** |
| ENSG00000230898 | AL596087.2 | 1.6487 | **0.0068** |
| ENSG00000231440 | AL358176.4 | 1.1268 | **0.0140** |
| ENSG00000231873 | AC099560.1 | 1.2498 | **0.0300** |
| ENSG00000231966 | AL359853.2 | 1.1414 | **0.0023** |
| ENSG00000232298 | AL138902.1 | 2.5261 | **0.0000** |
| ENSG00000232767 | AC016825.1 | 1.8189 | **0.0039** |
| ENSG00000232850 | AL590708.1 | 1.5305 | **0.0000** |
| ENSG00000232884 | AF127936.2 | 1.1172 | **0.0109** |
| ENSG00000232949 | AC002480.2 | 1.3852 | **0.0294** |
| ENSG00000233048 | LINC01722 | 1.3314 | **0.0456** |
| ENSG00000233421 | LINC01783 | 1.3941 | **0.0105** |
| ENSG00000233610 | LINC00462 | 1.1299 | **0.0392** |
| ENSG00000234571 | AC239798.2 | 1.4882 | **0.0011** |
| ENSG00000234665 | AL512625.3 | 1.1700 | **0.0349** |
| ENSG00000235820 | AL109935.1 | 2.1515 | **0.0006** |
| ENSG00000236242 | MYO16-AS1 | 1.0038 | **0.0041** |
| ENSG00000236384 | LINC00479 | 1.1674 | **0.0491** |
| ENSG00000236842 | AC010997.2 | 2.2017 | **0.0008** |
| ENSG00000236911 | AL137789.1 | 1.0654 | **0.0121** |
| ENSG00000237343 | AC246785.3 | 1.1645 | **0.0037** |
| ENSG00000237387 | AL022329.2 | 1.4498 | **0.0161** |
| ENSG00000237567 | Z97206.2 | 1.1686 | **0.0357** |
| ENSG00000239335 | LLPH-DT | 1.0464 | **0.0054** |
| ENSG00000240521 | AC092979.1 | 1.4313 | **0.0313** |
| ENSG00000241985 | WWTR1-IT1 | 1.3898 | **0.0183** |
| ENSG00000242611 | AC093627.6 | 1.1305 | **0.0382** |
| ENSG00000243810 | AL121721.1 | 1.2385 | **0.0428** |
| ENSG00000244247 | LINC01995 | 1.6772 | **0.0094** |
| ENSG00000244733 | AL132656.2 | 1.4018 | **0.0037** |
| ENSG00000245667 | AC006064.1 | 1.1696 | **0.0309** |
| ENSG00000246640 | PICART1 | 2.0852 | **0.0017** |
| ENSG00000249574 | AC226118.1 | 1.1129 | **0.0485** |
| ENSG00000249604 | AC096564.2 | 1.4000 | **0.0141** |
| ENSG00000249699 | LINC02261 | 1.4008 | **0.0161** |
| ENSG00000249753 | AC084357.2 | 1.5284 | **0.0167** |
| ENSG00000251018 | HMMR-AS1 | 1.3890 | **0.0196** |
| ENSG00000251209 | LINC00923 | 1.7070 | **0.0040** |
| ENSG00000251665 | AC005920.3 | 1.7605 | **0.0061** |
| ENSG00000253519 | AC106801.1 | 2.2136 | **0.0011** |
| ENSG00000253809 | AL160262.1 | 1.6458 | **0.0101** |
| ENSG00000253851 | AC025370.1 | 1.4393 | **0.0175** |
| ENSG00000254287 | AC007991.4 | 1.4735 | **0.0144** |
| ENSG00000254859 | AC067930.3 | 1.1261 | **0.0092** |
| ENSG00000255136 | AP001972.3 | 1.4400 | **0.0135** |
| ENSG00000255160 | AC009652.1 | 1.2656 | **0.0262** |
| ENSG00000255314 | AC024475.4 | 1.1125 | **0.0000** |
| ENSG00000255476 | AC011092.2 | 1.2901 | **0.0293** |
| ENSG00000256813 | AP000777.3 | 1.2805 | **0.0033** |
| ENSG00000258379 | AL355097.1 | 1.3410 | **0.0298** |
| ENSG00000258857 | AL359397.1 | 1.9107 | **0.0055** |
| ENSG00000259130 | AL133371.3 | 2.5614 | **0.0002** |
| ENSG00000259519 | AC051619.4 | 1.6401 | **0.0036** |
| ENSG00000259793 | AC013726.1 | 1.0934 | **0.0026** |
| ENSG00000260235 | AC105020.3 | 1.0823 | **0.0190** |
| ENSG00000260495 | AC009148.1 | 1.1148 | **0.0000** |
| ENSG00000260871 | AC093510.2 | 1.0826 | **0.0188** |
| ENSG00000260971 | AC119674.1 | 1.6087 | **0.0152** |
| ENSG00000261268 | AC112236.1 | 1.3838 | **0.0140** |
| ENSG00000261798 | AL033527.3 | 1.0199 | **0.0102** |
| ENSG00000262521 | AJ003147.1 | 1.3225 | **0.0367** |
| ENSG00000263412 | AC004477.1 | 1.0077 | **0.0000** |
| ENSG00000263745 | AP005230.1 | 1.9632 | **0.0024** |
| ENSG00000264421 | AC007448.2 | 1.7500 | **0.0061** |
| ENSG00000264735 | AC145207.7 | 1.6306 | **0.0101** |
| ENSG00000265282 | AC005828.4 | 1.0343 | **0.0118** |
| ENSG00000265478 | AC107982.3 | 1.0067 | **0.0000** |
| ENSG00000265542 | AC015845.2 | 1.5620 | **0.0056** |
| ENSG00000267024 | AC008747.1 | 1.4145 | **0.0214** |
| ENSG00000267054 | AC008738.1 | 1.0391 | **0.0213** |
| ENSG00000267219 | AC010504.1 | 1.3649 | **0.0010** |
| ENSG00000267240 | AC011524.2 | 2.3986 | **0.0001** |
| ENSG00000267328 | AC002398.2 | 1.0396 | **0.0111** |
| ENSG00000267452 | LINC02073 | 1.0067 | **0.0437** |
| ENSG00000267568 | AC016168.2 | 1.2208 | **0.0028** |
| ENSG00000267662 | AC025809.1 | 1.0819 | **0.0274** |
| ENSG00000267762 | AC048380.1 | 1.2420 | **0.0428** |
| ENSG00000267808 | AC018755.1 | 1.1695 | **0.0148** |
| ENSG00000267857 | AL133499.1 | 1.1564 | **0.0448** |
| ENSG00000269392 | AC008655.2 | 1.0145 | **0.0424** |
| ENSG00000269400 | AC008734.2 | 1.5698 | **0.0099** |
| ENSG00000269489 | AL589765.6 | 1.0328 | **0.0331** |
| ENSG00000269859 | AC008735.3 | 1.3113 | **0.0277** |
| ENSG00000270001 | AL121894.2 | 1.0690 | **0.0000** |
| ENSG00000270104 | AL670729.3 | 1.0194 | **0.0006** |
| ENSG00000270133 | AC025766.1 | 1.0050 | **0.0000** |
| ENSG00000270210 | AC104695.3 | 1.2426 | **0.0000** |
| ENSG00000270557 | AC013731.1 | 1.1135 | **0.0000** |
| ENSG00000270607 | AC009549.1 | 1.2625 | **0.0428** |
| ENSG00000271259 | AC010201.1 | 1.0931 | **0.0006** |
| ENSG00000271653 | AC097359.3 | 1.2247 | **0.0410** |
| ENSG00000271952 | LINC01954 | 3.0486 | **0.0001** |
| ENSG00000271993 | AC126118.1 | 1.0708 | **0.0001** |
| ENSG00000272071 | AC122710.2 | 1.4507 | **0.0183** |
| ENSG00000272783 | AC147067.2 | 1.3645 | **0.0093** |
| ENSG00000272810 | U91328.3 | 1.4445 | **0.0008** |
| ENSG00000272862 | AC106052.1 | 1.1317 | **0.0394** |
| ENSG00000273248 | AC010997.5 | 2.0282 | **0.0001** |
| ENSG00000273258 | AC016737.2 | 1.1426 | **0.0001** |
| ENSG00000273287 | AL008718.2 | 1.3568 | **0.0292** |
| ENSG00000273313 | RBAKDN | 1.1709 | **0.0388** |
| ENSG00000274340 | AC032011.1 | 1.1490 | **0.0250** |
| ENSG00000274560 | AC010205.1 | 1.4306 | **0.0152** |
| ENSG00000274698 | AC099521.1 | 1.0413 | **0.0478** |
| ENSG00000274719 | AC012653.2 | 1.2780 | **0.0428** |
| ENSG00000275248 | AL355810.1 | 1.5610 | **0.0152** |
| ENSG00000275327 | AL354950.2 | 1.3956 | **0.0403** |
| ENSG00000275542 | AC027601.3 | 1.2031 | **0.0190** |
| ENSG00000275649 | AL445584.2 | 1.4478 | **0.0045** |
| ENSG00000276509 | AC239799.1 | 1.0606 | **0.0393** |
| ENSG00000276693 | AC138466.3 | 2.1317 | **0.0006** |
| ENSG00000276742 | AL731566.2 | 2.0108 | **0.0004** |
| ENSG00000278719 | MCM8-AS1 | 1.3264 | **0.0057** |
| ENSG00000278982 | AL139125.1 | 1.2299 | **0.0457** |
| ENSG00000279154 | AL353726.2 | 1.4440 | **0.0274** |
| ENSG00000279360 | AC007546.2 | 1.5154 | **0.0046** |
| ENSG00000279404 | AC008739.5 | 1.3931 | **0.0161** |
| ENSG00000279518 | AC083843.4 | 1.1679 | **0.0002** |
| ENSG00000279660 | AC005703.6 | 1.8284 | **0.0037** |
| ENSG00000279693 | AC099521.2 | 1.0851 | **0.0000** |
| ENSG00000279715 | AL161912.4 | 1.7836 | **0.0026** |
| ENSG00000279719 | AC015871.5 | 1.0274 | **0.0029** |
| ENSG00000279786 | AC105235.1 | 1.1455 | **0.0015** |
| ENSG00000280076 | AC112693.3 | 1.5247 | **0.0167** |
| ENSG00000280152 | AC009078.3 | 1.3094 | **0.0360** |
| ENSG00000280231 | AL031719.2 | 1.0565 | **0.0021** |
| ENSG00000280242 | AL450226.2 | 2.1170 | **0.0013** |
| ENSG00000280259 | AL512662.1 | 1.5599 | **0.0044** |
| ENSG00000280382 | AC096920.1 | 1.2045 | **0.0000** |
| ENSG00000280395 | AL034546.1 | 1.2480 | **0.0428** |
| ENSG00000282742 | AC093323.3 | 1.1345 | **0.0001** |
| ENSG00000282907 | Z98883.1 | 1.8364 | **0.0017** |
| ENSG00000283061 | AC215522.3 | 1.5134 | **0.0163** |
| ENSG00000283098 | AL132857.1 | 1.4239 | **0.0217** |
| ENSG00000283155 | AC116353.4 | 1.2894 | **0.0020** |
| ENSG00000283341 | AC068205.2 | 1.0532 | **0.0000** |
| ENSG00000283352 | AL021368.4 | 1.1148 | **0.0425** |
| ENSG00000283579 | AL160035.1 | 1.2898 | **0.0395** |
| ENSG00000284966 | AL138689.2 | 1.0910 | **0.0413** |
| ENSG00000284999 | AL591518.1 | 2.5598 | **0.0007** |
| ENSG00000285630 | AL590068.3 | 1.2772 | **0.0467** |
| ENSG00000285639 | AL133259.1 | 1.7024 | **0.0100** |
| ENSG00000285647 | AL671883.3 | 1.5405 | **0.0258** |
| ENSG00000285771 | AL139095.5 | 1.7519 | **0.0061** |
| ENSG00000285904 | AC006452.1 | 1.4643 | **0.0178** |
| ENSG00000285933 | AP003498.2 | 1.0513 | **0.0013** |
| ENSG00000286189 | AC108021.1 | 1.1898 | **0.0172** |
| ENSG00000124915 | AP002380.1 | -1.5320 | **0.0114** |
| ENSG00000186594 | MIR22HG | -1.1399 | **0.0001** |
| ENSG00000198454 | PRR31 | -1.2718 | **0.0133** |
| ENSG00000204709 | LINC01556 | -1.1579 | **0.0392** |
| ENSG00000204832 | ST8SIA6-AS1 | -1.5403 | **0.0092** |
| ENSG00000214043 | LINC02347 | -1.3925 | **0.0179** |
| ENSG00000218416 | AC110619.1 | -1.4811 | **0.0103** |
| ENSG00000223662 | SAMSN1-AS1 | -1.3584 | **0.0099** |
| ENSG00000224077 | AP000936.1 | -1.7596 | **0.0025** |
| ENSG00000225884 | AC098872.1 | -1.3500 | **0.0227** |
| ENSG00000226644 | AL121899.1 | -1.3645 | **0.0150** |
| ENSG00000226851 | AC004112.1 | -1.1967 | **0.0436** |
| ENSG00000227237 | AL672291.1 | -1.3520 | **0.0042** |
| ENSG00000227386 | AC091705.1 | -1.4374 | **0.0229** |
| ENSG00000227421 | LINC01724 | -1.3186 | **0.0323** |
| ENSG00000227591 | HSD11B1-AS1 | -1.0823 | **0.0000** |
| ENSG00000227660 | UST-AS1 | -1.7773 | **0.0026** |
| ENSG00000227712 | AL359915.1 | -1.6544 | **0.0044** |
| ENSG00000228065 | LINC01515 | -1.9592 | **0.0006** |
| ENSG00000228170 | AL031963.1 | -1.3558 | **0.0171** |
| ENSG00000228275 | ARMCX3-AS1 | -1.4618 | **0.0001** |
| ENSG00000228386 | AL031668.1 | -1.5819 | **0.0004** |
| ENSG00000228763 | LIMS1-AS1 | -1.3183 | **0.0326** |
| ENSG00000229188 | AL050349.1 | -1.1745 | **0.0326** |
| ENSG00000229740 | U91324.1 | -1.3411 | **0.0153** |
| ENSG00000229867 | STEAP3-AS1 | -1.0415 | **0.0028** |
| ENSG00000230392 | AC004835.1 | -1.5219 | **0.0095** |
| ENSG00000230536 | AL360268.1 | -1.5635 | **0.0034** |
| ENSG00000230537 | AL158071.3 | -1.0509 | **0.0000** |
| ENSG00000230731 | AL589745.1 | -1.2790 | **0.0327** |
| ENSG00000230848 | AL360268.2 | -1.3907 | **0.0179** |
| ENSG00000231140 | BX005214.2 | -1.3358 | **0.0272** |
| ENSG00000231194 | FARP1-AS1 | -1.2005 | **0.0237** |
| ENSG00000231476 | AC074389.2 | -1.3354 | **0.0106** |
| ENSG00000231851 | AC104655.1 | -1.2003 | **0.0021** |
| ENSG00000231896 | AC019185.2 | -1.5100 | **0.0123** |
| ENSG00000231943 | PGM5P4-AS1 | -1.5140 | **0.0123** |
| ENSG00000232396 | AC004882.1 | -1.4883 | **0.0108** |
| ENSG00000232581 | AC079742.1 | -1.0695 | **0.0053** |
| ENSG00000232909 | AL157823.2 | -1.8234 | **0.0014** |
| ENSG00000232998 | VPS13A-AS1 | -1.3088 | **0.0058** |
| ENSG00000233643 | LINC02625 | -1.4528 | **0.0083** |
| ENSG00000233993 | AL121900.1 | -1.3277 | **0.0045** |
| ENSG00000234022 | AC008278.2 | -1.2152 | **0.0085** |
| ENSG00000235185 | AL031727.2 | -1.3011 | **0.0227** |
| ENSG00000235728 | AC007349.3 | -1.6304 | **0.0034** |
| ENSG00000236069 | AL136987.1 | -2.0223 | **0.0007** |
| ENSG00000237224 | AL157832.2 | -1.3673 | **0.0241** |
| ENSG00000237232 | ZNF295-AS1 | -1.1941 | **0.0019** |
| ENSG00000237371 | AL355803.1 | -1.5590 | **0.0077** |
| ENSG00000237585 | LINC00407 | -1.3396 | **0.0258** |
| ENSG00000240093 | AC093627.3 | -1.1784 | **0.0414** |
| ENSG00000245059 | AC092718.1 | -1.0694 | **0.0033** |
| ENSG00000245526 | LINC00461 | -1.6592 | **0.0106** |
| ENSG00000245688 | AC008659.1 | -1.1825 | **0.0278** |
| ENSG00000246740 | PLA2G4E-AS1 | -1.2081 | **0.0261** |
| ENSG00000246777 | AC044802.1 | -1.2673 | **0.0424** |
| ENSG00000248607 | AC117422.1 | -1.5144 | **0.0123** |
| ENSG00000248991 | AC097375.1 | -1.2187 | **0.0025** |
| ENSG00000249706 | AC105384.1 | -1.9665 | **0.0005** |
| ENSG00000250282 | AC002401.2 | -1.5126 | **0.0109** |
| ENSG00000250739 | LINC01262 | -1.1261 | **0.0220** |
| ENSG00000251183 | LINC01861 | -1.2514 | **0.0298** |
| ENSG00000251391 | AC113378.1 | -1.1775 | **0.0412** |
| ENSG00000251611 | FAM160A1-DT | -1.0677 | **0.0134** |
| ENSG00000253125 | AC055854.1 | -1.6832 | **0.0002** |
| ENSG00000253666 | AP000424.1 | -1.1587 | **0.0282** |
| ENSG00000253811 | AC136424.2 | -1.5238 | **0.0123** |
| ENSG00000254433 | AP001001.1 | -1.0888 | **0.0366** |
| ENSG00000254538 | AC027018.1 | -1.3452 | **0.0145** |
| ENSG00000254813 | AC123777.1 | -1.0891 | **0.0388** |
| ENSG00000255548 | AP003043.1 | -1.2714 | **0.0350** |
| ENSG00000255649 | AC008114.1 | -1.1848 | **0.0179** |
| ENSG00000256512 | AC009511.1 | -1.1752 | **0.0423** |
| ENSG00000256577 | AC007406.4 | -1.5133 | **0.0074** |
| ENSG00000256955 | AC131009.2 | -1.8248 | **0.0020** |
| ENSG00000257000 | AC137590.1 | -1.0517 | **0.0386** |
| ENSG00000257023 | AC087241.3 | -1.6435 | **0.0033** |
| ENSG00000257568 | AC079035.1 | -1.4737 | **0.0123** |
| ENSG00000257657 | AC079950.1 | -1.1735 | **0.0298** |
| ENSG00000257955 | AC004801.3 | -2.4301 | **0.0000** |
| ENSG00000258038 | LINC02327 | -1.2760 | **0.0453** |
| ENSG00000258413 | AL158801.2 | -1.3370 | **0.0001** |
| ENSG00000258455 | AL158801.3 | -1.1156 | **0.0497** |
| ENSG00000258512 | LINC00239 | -1.1555 | **0.0000** |
| ENSG00000258657 | AL136018.1 | -1.1032 | **0.0054** |
| ENSG00000258673 | LINC01397 | -1.5059 | **0.0098** |
| ENSG00000258807 | AL359237.1 | -1.3064 | **0.0214** |
| ENSG00000259202 | AC012568.1 | -1.0879 | **0.0255** |
| ENSG00000259269 | AC109630.1 | -1.4551 | **0.0118** |
| ENSG00000259390 | AC022196.1 | -1.2056 | **0.0341** |
| ENSG00000259438 | MAPK6-DT | -1.5072 | **0.0090** |
| ENSG00000260118 | AL157700.1 | -1.1287 | **0.0479** |
| ENSG00000260123 | AC013565.1 | -1.1126 | **0.0311** |
| ENSG00000260351 | AC024270.2 | -1.0980 | **0.0327** |
| ENSG00000260420 | LINC02182 | -1.5086 | **0.0090** |
| ENSG00000260430 | AC099518.1 | -1.2320 | **0.0263** |
| ENSG00000260578 | AC110597.1 | -1.1781 | **0.0282** |
| ENSG00000260613 | Z98885.2 | -1.1855 | **0.0264** |
| ENSG00000261026 | AC105046.1 | -2.6590 | **0.0004** |
| ENSG00000261068 | AL512274.1 | -1.4025 | **0.0134** |
| ENSG00000261407 | AC013565.3 | -1.2405 | **0.0001** |
| ENSG00000261592 | AC010531.3 | -1.2041 | **0.0171** |
| ENSG00000262006 | AC005920.4 | -1.4566 | **0.0098** |
| ENSG00000262202 | AC007952.4 | -1.2423 | **0.0038** |
| ENSG00000262223 | AC110285.1 | -1.1209 | **0.0113** |
| ENSG00000262477 | AC021224.1 | -1.0439 | **0.0475** |
| ENSG00000262772 | LINC01977 | -1.4058 | **0.0130** |
| ENSG00000263393 | AC011825.2 | -1.3266 | **0.0227** |
| ENSG00000264019 | AC018521.2 | -1.0192 | **0.0277** |
| ENSG00000264491 | AC005544.1 | -1.4235 | **0.0135** |
| ENSG00000265257 | AP005263.1 | -1.0414 | **0.0074** |
| ENSG00000265511 | AC020558.2 | -1.1017 | **0.0243** |
| ENSG00000266100 | AC007431.2 | -1.3811 | **0.0179** |
| ENSG00000267257 | AC105105.1 | -1.2113 | **0.0065** |
| ENSG00000267299 | AC011444.3 | -1.4306 | **0.0100** |
| ENSG00000267313 | AC021504.1 | -1.7838 | **0.0033** |
| ENSG00000267496 | FAM215A | -1.4215 | **0.0082** |
| ENSG00000267503 | AP005131.3 | -1.1029 | **0.0327** |
| ENSG00000267550 | AC022517.1 | -1.2684 | **0.0076** |
| ENSG00000267582 | AC020916.2 | -1.3849 | **0.0179** |
| ENSG00000267705 | AC104365.3 | -1.1511 | **0.0273** |
| ENSG00000268480 | LINC01862 | -1.4396 | **0.0152** |
| ENSG00000269688 | AC008982.2 | -1.0147 | **0.0000** |
| ENSG00000269926 | DDIT4-AS1 | -1.2263 | **0.0000** |
| ENSG00000269927 | AC004817.3 | -1.3056 | **0.0006** |
| ENSG00000270761 | AL355353.1 | -1.0454 | **0.0311** |
| ENSG00000270816 | LINC00221 | -1.2215 | **0.0251** |
| ENSG00000270996 | AC005034.4 | -1.3249 | **0.0220** |
| ENSG00000271127 | AP000526.1 | -1.1664 | **0.0476** |
| ENSG00000271787 | AC104794.4 | -1.1562 | **0.0408** |
| ENSG00000271792 | AC008667.4 | -1.1227 | **0.0480** |
| ENSG00000272128 | AP006545.2 | -1.1087 | **0.0480** |
| ENSG00000272298 | AC091544.6 | -1.2477 | **0.0350** |
| ENSG00000272384 | AC016405.3 | -1.0332 | **0.0232** |
| ENSG00000272396 | AC005392.3 | -1.2460 | **0.0426** |
| ENSG00000272695 | GAS6-DT | -1.4173 | **0.0179** |
| ENSG00000272912 | AL356608.1 | -1.1577 | **0.0289** |
| ENSG00000273172 | LINC02091 | -1.4158 | **0.0179** |
| ENSG00000273214 | AL031587.4 | -1.2300 | **0.0350** |
| ENSG00000273257 | AC069200.1 | -1.4133 | **0.0119** |
| ENSG00000273338 | AC103591.3 | -1.3091 | **0.0015** |
| ENSG00000273396 | LINC01396 | -1.2253 | **0.0365** |
| ENSG00000273403 | AC107294.3 | -1.1616 | **0.0482** |
| ENSG00000273428 | AC004832.6 | -2.2989 | **0.0000** |
| ENSG00000273796 | BX322562.1 | -1.4104 | **0.0158** |
| ENSG00000274198 | AC025521.1 | -1.2228 | **0.0468** |
| ENSG00000274825 | AL023803.2 | -1.2354 | **0.0357** |
| ENSG00000275265 | AC127002.1 | -1.3656 | **0.0174** |
| ENSG00000276107 | AC037198.1 | -1.3307 | **0.0088** |
| ENSG00000277287 | AL109976.1 | -1.1870 | **0.0209** |
| ENSG00000277382 | AC005837.3 | -1.2218 | **0.0312** |
| ENSG00000278969 | AC026310.3 | -1.6180 | **0.0050** |
| ENSG00000278990 | AL132796.3 | -1.1633 | **0.0020** |
| ENSG00000279070 | AC073263.2 | -3.0210 | **0.0000** |
| ENSG00000279393 | AL139005.1 | -1.9112 | **0.0022** |
| ENSG00000279480 | AC138466.4 | -1.0015 | **0.0359** |
| ENSG00000279658 | AC110491.2 | -1.5961 | **0.0030** |
| ENSG00000279812 | AC120057.4 | -1.2207 | **0.0211** |
| ENSG00000279949 | AC022784.6 | -1.6457 | **0.0025** |
| ENSG00000280441 | FP236383.1 | -1.5800 | **0.0000** |
| ENSG00000282022 | AC006581.2 | -1.8439 | **0.0030** |
| ENSG00000283096 | AL731555.1 | -1.3827 | **0.0187** |
| ENSG00000283314 | LINC02357 | -1.4687 | **0.0207** |
| ENSG00000284600 | AC093390.2 | -1.3763 | **0.0179** |
| ENSG00000284618 | AL391294.1 | -1.2440 | **0.0373** |
| ENSG00000285467 | AL136419.1 | -1.1499 | **0.0442** |
| ENSG00000285766 | AC092042.4 | -1.6532 | **0.0058** |
| ENSG00000285856 | AL353704.1 | -1.6519 | **0.0004** |
| ENSG00000286229 | AL133335.1 | -1.3187 | **0.0264** |

The bold text indicates significance.

**Table S3** mRNAs differentially expressed between healthy controls and AR.

| ENSG ID | Gene symbol | log_2-_fold change | *P* |
| --- | --- | --- | --- |
| ENSG00000002726 | AOC1 | 1.5074 | **0.0030** |
| ENSG00000007372 | PAX6 | 1.3996 | **0.0024** |
| ENSG00000017427 | IGF1 | 1.1786 | **0.0198** |
| ENSG00000039139 | DNAH5 | 1.8986 | **0.0007** |
| ENSG00000046889 | PREX2 | 1.3124 | **0.0260** |
| ENSG00000048462 | TNFRSF17 | 1.0426 | **0.0003** |
| ENSG00000056736 | IL17RB | 1.1296 | **0.0004** |
| ENSG00000075891 | PAX2 | 1.4142 | **0.0122** |
| ENSG00000077279 | DCX | 2.0235 | **0.0045** |
| ENSG00000077616 | NAALAD2 | 1.1525 | **0.0037** |
| ENSG00000078295 | ADCY2 | 1.2964 | **0.0332** |
| ENSG00000079112 | CDH17 | 1.7980 | **0.0045** |
| ENSG00000081248 | CACNA1S | 1.5134 | **0.0130** |
| ENSG00000082684 | SEMA5B | 1.3341 | **0.0201** |
| ENSG00000088926 | F11 | 1.0860 | **0.0327** |
| ENSG00000090402 | SI | 1.6060 | **0.0178** |
| ENSG00000092067 | CEBPE | 1.3161 | **0.0000** |
| ENSG00000092068 | SLC7A8 | 1.0362 | **0.0000** |
| ENSG00000095596 | CYP26A1 | 1.2114 | **0.0409** |
| ENSG00000095777 | MYO3A | 1.2864 | **0.0119** |
| ENSG00000100053 | CRYBB3 | 1.1098 | **0.0059** |
| ENSG00000101311 | FERMT1 | 1.6406 | **0.0031** |
| ENSG00000103056 | SMPD3 | 1.0346 | **0.0001** |
| ENSG00000103089 | FA2H | 1.1249 | **0.0025** |
| ENSG00000103355 | PRSS33 | 1.1626 | **0.0002** |
| ENSG00000103855 | CD276 | 2.1346 | **0.0001** |
| ENSG00000104067 | TJP1 | 1.7503 | **0.0246** |
| ENSG00000104112 | SCG3 | 1.1850 | **0.0405** |
| ENSG00000105205 | CLC | 1.1389 | **0.0000** |
| ENSG00000105357 | MYH14 | 3.9146 | **0.0004** |
| ENSG00000105366 | SIGLEC8 | 1.4028 | **0.0000** |
| ENSG00000105976 | MET | 2.1721 | **0.0000** |
| ENSG00000108702 | CCL1 | 1.8267 | **0.0029** |
| ENSG00000109099 | PMP22 | 1.5346 | **0.0001** |
| ENSG00000111291 | GPRC5D | 1.1306 | **0.0039** |
| ENSG00000112759 | SLC29A1 | 1.1560 | **0.0000** |
| ENSG00000113361 | CDH6 | 2.0976 | **0.0025** |
| ENSG00000115008 | IL1A | 2.5379 | **0.0081** |
| ENSG00000115009 | CCL20 | 2.2185 | **0.0031** |
| ENSG00000115361 | ACADL | 1.3542 | **0.0365** |
| ENSG00000115602 | IL1RL1 | 1.2879 | **0.0000** |
| ENSG00000119125 | GDA | 1.5864 | **0.0101** |
| ENSG00000120693 | SMAD9 | 1.5731 | **0.0070** |
| ENSG00000121361 | KCNJ8 | 1.4075 | **0.0187** |
| ENSG00000122691 | TWIST1 | 1.1900 | **0.0405** |
| ENSG00000123119 | NECAB1 | 1.1169 | **0.0228** |
| ENSG00000124227 | ANKRD60 | 1.2010 | **0.0405** |
| ENSG00000128045 | RASL11B | 1.5909 | **0.0128** |
| ENSG00000129991 | TNNI3 | 2.0523 | **0.0018** |
| ENSG00000130054 | FAM155B | 1.4285 | **0.0353** |
| ENSG00000134757 | DSG3 | 1.1867 | **0.0016** |
| ENSG00000134874 | DZIP1 | 1.5590 | **0.0057** |
| ENSG00000136872 | ALDOB | 1.5129 | **0.0130** |
| ENSG00000137225 | CAPN11 | 1.0379 | **0.0132** |
| ENSG00000137285 | TUBB2B | 1.6695 | **0.0177** |
| ENSG00000138161 | CUZD1 | 1.2507 | **0.0017** |
| ENSG00000138395 | CDK15 | 1.6656 | **0.0000** |
| ENSG00000138587 | MNS1 | 1.0327 | **0.0208** |
| ENSG00000138792 | ENPEP | 1.4443 | **0.0076** |
| ENSG00000140067 | FAM181A | 1.3349 | **0.0365** |
| ENSG00000140459 | CYP11A1 | 1.3303 | **0.0028** |
| ENSG00000140945 | CDH13 | 1.5737 | **0.0054** |
| ENSG00000144278 | GALNT13 | 1.1542 | **0.0381** |
| ENSG00000146469 | VIP | 1.1954 | **0.0477** |
| ENSG00000147246 | HTR2C | 2.0613 | **0.0009** |
| ENSG00000147697 | GSDMC | 1.2089 | **0.0052** |
| ENSG00000148344 | PTGES | 1.4631 | **0.0061** |
| ENSG00000149735 | GPHA2 | 1.2500 | **0.0246** |
| ENSG00000150275 | PCDH15 | 3.0734 | **0.0000** |
| ENSG00000150893 | FREM2 | 1.4607 | **0.0204** |
| ENSG00000151388 | ADAMTS12 | 2.3273 | **0.0017** |
| ENSG00000152591 | DSPP | 1.5903 | **0.0128** |
| ENSG00000152672 | CLEC4F | 1.0024 | **0.0340** |
| ENSG00000154646 | TMPRSS15 | 1.4381 | **0.0304** |
| ENSG00000158315 | RHBDL2 | 1.4833 | **0.0217** |
| ENSG00000159261 | CLDN14 | 1.8786 | **0.0028** |
| ENSG00000160180 | TFF3 | 1.2759 | **0.0005** |
| ENSG00000160396 | HIPK4 | 1.0027 | **0.0417** |
| ENSG00000160882 | CYP11B1 | 1.6382 | **0.0076** |
| ENSG00000161905 | ALOX15 | 1.1633 | **0.0001** |
| ENSG00000162598 | C1orf87 | 1.3378 | **0.0251** |
| ENSG00000163053 | SLC16A14 | 1.0980 | **0.0001** |
| ENSG00000163380 | LMOD3 | 1.1231 | **0.0453** |
| ENSG00000164176 | EDIL3 | 1.4685 | **0.0343** |
| ENSG00000164591 | MYOZ3 | 1.1927 | **0.0108** |
| ENSG00000164932 | CTHRC1 | 1.1268 | **0.0332** |
| ENSG00000165091 | TMC1 | 1.7488 | **0.0004** |
| ENSG00000165204 | OR1K1 | 1.2784 | **0.0356** |
| ENSG00000165863 | C10orf82 | 1.1681 | **0.0020** |
| ENSG00000166984 | TCP10L2 | 1.0935 | **0.0023** |
| ENSG00000167646 | DNAAF3 | 1.0802 | **0.0272** |
| ENSG00000167807 | AC011511.1 | 1.1734 | **0.0329** |
| ENSG00000168309 | FAM107A | 1.6742 | **0.0075** |
| ENSG00000168356 | SCN11A | 1.3182 | **0.0156** |
| ENSG00000168658 | VWA3B | 1.1556 | **0.0156** |
| ENSG00000168779 | SHOX2 | 1.0951 | **0.0020** |
| ENSG00000169071 | ROR2 | 1.0487 | **0.0481** |
| ENSG00000169126 | ARMC4 | 1.9755 | **0.0039** |
| ENSG00000169575 | VPREB1 | 1.2066 | **0.0442** |
| ENSG00000170044 | ZPLD1 | 1.1750 | **0.0416** |
| ENSG00000170122 | FOXD4 | 1.0387 | **0.0011** |
| ENSG00000170500 | LONRF2 | 1.0376 | **0.0011** |
| ENSG00000170927 | PKHD1 | 1.7468 | **0.0006** |
| ENSG00000171116 | HSFX1 | 1.8975 | **0.0021** |
| ENSG00000171759 | PAH | 1.4109 | **0.0172** |
| ENSG00000173714 | WFIKKN2 | 2.2394 | **0.0003** |
| ENSG00000173867 | AC013489.1 | 1.0776 | **0.0036** |
| ENSG00000174567 | GOLT1A | 1.3498 | **0.0098** |
| ENSG00000174950 | CD164L2 | 1.6486 | **0.0072** |
| ENSG00000175315 | CST6 | 2.6571 | **0.0013** |
| ENSG00000177181 | RIMKLA | 1.0937 | **0.0012** |
| ENSG00000177807 | KCNJ10 | 1.0012 | **0.0431** |
| ENSG00000178233 | TMEM151B | 1.3038 | **0.0337** |
| ENSG00000178343 | SHISA3 | 1.4253 | **0.0147** |
| ENSG00000178445 | GLDC | 1.0261 | **0.0092** |
| ENSG00000178462 | TUBAL3 | 1.5021 | **0.0131** |
| ENSG00000179873 | NLRP11 | 1.7596 | **0.0043** |
| ENSG00000180697 | C3orf22 | 1.0059 | **0.0458** |
| ENSG00000181625 | SLX1B | 1.2765 | **0.0223** |
| ENSG00000182035 | ADIG | 1.4321 | **0.0013** |
| ENSG00000182771 | GRID1 | 1.0361 | **0.0105** |
| ENSG00000183379 | SYNDIG1L | 1.9466 | **0.0006** |
| ENSG00000183844 | FAM3B | 4.9694 | **0.0000** |
| ENSG00000184611 | KCNH7 | 1.5495 | **0.0002** |
| ENSG00000185155 | MIXL1 | 1.4327 | **0.0124** |
| ENSG00000185290 | NUPR2 | 1.1312 | **0.0495** |
| ENSG00000185774 | KCNIP4 | 1.1861 | **0.0444** |
| ENSG00000186732 | MPPED1 | 1.6655 | **0.0048** |
| ENSG00000187537 | POTEG | 1.3125 | **0.0339** |
| ENSG00000187559 | FOXD4L3 | 1.2486 | **0.0094** |
| ENSG00000188060 | RAB42 | 1.2211 | **0.0056** |
| ENSG00000188076 | SCGB1C1 | 1.0598 | **0.0274** |
| ENSG00000188162 | OTOG | 2.0693 | **0.0012** |
| ENSG00000189068 | VSTM1 | 1.0157 | **0.0000** |
| ENSG00000189184 | PCDH18 | 1.2755 | **0.0319** |
| ENSG00000189292 | ALKAL2 | 1.2703 | **0.0010** |
| ENSG00000197565 | COL4A6 | 1.6519 | **0.0048** |
| ENSG00000198010 | DLGAP2 | 2.6864 | **0.0049** |
| ENSG00000198128 | OR2L3 | 1.1415 | **0.0151** |
| ENSG00000204511 | MCCD1 | 1.2553 | **0.0349** |
| ENSG00000204882 | GPR20 | 1.0059 | **0.0348** |
| ENSG00000204928 | GRXCR2 | 1.2563 | **0.0355** |
| ENSG00000205436 | EXOC3L4 | 1.3101 | **0.0382** |
| ENSG00000205439 | KRTAP12-3 | 1.2453 | **0.0374** |
| ENSG00000205927 | OLIG2 | 1.4453 | **0.0000** |
| ENSG00000215277 | RNF212B | 1.4563 | **0.0003** |
| ENSG00000235961 | PNMA6A | 1.9656 | **0.0016** |
| ENSG00000237988 | OR2I1P | 1.1302 | **0.0001** |
| ENSG00000241322 | CDRT1 | 1.2703 | **0.0211** |
| ENSG00000244115 | DNAJC25-GNG10 | 1.1109 | **0.0384** |
| ENSG00000254709 | IGLL5 | 1.3690 | **0.0000** |
| ENSG00000255221 | CARD17 | 1.0196 | **0.0012** |
| ENSG00000256222 | MTRNR2L3 | 1.3636 | **0.0387** |
| ENSG00000256374 | PPIAL4D | 1.6503 | **0.0222** |
| ENSG00000256646 | AC010132.3 | 1.1749 | **0.0178** |
| ENSG00000257138 | TAS2R38 | 1.3085 | **0.0132** |
| ENSG00000261678 | SCRT1 | 1.6913 | **0.0076** |
| ENSG00000265690 | AC074143.2 | 1.9391 | **0.0021** |
| ENSG00000269242 | AC010422.3 | 1.0568 | **0.0097** |
| ENSG00000273513 | TBC1D3K | 1.4037 | **0.0291** |
| ENSG00000274276 | CBSL | 1.1411 | **0.0331** |
| ENSG00000274529 | SEBOX | 1.2506 | **0.0080** |
| ENSG00000274736 | CCL23 | 2.2485 | **0.0001** |
| ENSG00000274791 | F8A2 | 1.4592 | **0.0176** |
| ENSG00000276409 | CCL14 | 1.3110 | **0.0330** |
| ENSG00000279263 | OR2L8 | 1.9194 | **0.0001** |
| ENSG00000280094 | OR1B1 | 1.2860 | **0.0263** |
| ENSG00000280969 | RPS4Y2 | 1.5406 | **0.0349** |
| ENSG00000284194 | SCO2 | 3.1620 | **0.0012** |
| ENSG00000284356 | TAF11L10 | 1.2687 | **0.0438** |
| ENSG00000284686 | AC119674.2 | 1.3159 | **0.0330** |
| ENSG00000285708 | AC097634.4 | 1.2992 | **0.0246** |
| ENSG00000286019 | AC239811.1 | 1.3031 | **0.0330** |
| ENSG00000036448 | MYOM2 | -1.1350 | **0.0404** |
| ENSG00000054938 | CHRDL2 | -1.4393 | **0.0162** |
| ENSG00000057593 | F7 | -1.3336 | **0.0072** |
| ENSG00000061656 | SPAG4 | -1.1519 | **0.0005** |
| ENSG00000067842 | ATP2B3 | -1.2699 | **0.0223** |
| ENSG00000073756 | PTGS2 | -1.6366 | **0.0000** |
| ENSG00000076864 | RAP1GAP | -1.4814 | **0.0037** |
| ENSG00000077942 | FBLN1 | -2.0024 | **0.0049** |
| ENSG00000078114 | NEBL | -1.5088 | **0.0010** |
| ENSG00000089199 | CHGB | -1.2654 | **0.0393** |
| ENSG00000090104 | RGS1 | -1.2272 | **0.0005** |
| ENSG00000100565 | LRRC74A | -1.2180 | **0.0478** |
| ENSG00000101203 | COL20A1 | -1.3508 | **0.0050** |
| ENSG00000102466 | FGF14 | -1.6213 | **0.0200** |
| ENSG00000104321 | TRPA1 | -1.6379 | **0.0020** |
| ENSG00000105695 | MAG | -1.0873 | **0.0202** |
| ENSG00000106571 | GLI3 | -1.1566 | **0.0014** |
| ENSG00000109321 | AREG | -1.8235 | **0.0002** |
| ENSG00000109758 | HGFAC | -1.2712 | **0.0243** |
| ENSG00000110848 | CD69 | -1.2949 | **0.0000** |
| ENSG00000112852 | PCDHB2 | -1.4515 | **0.0123** |
| ENSG00000113070 | HBEGF | -1.4430 | **0.0013** |
| ENSG00000115414 | FN1 | -2.5607 | **0.0000** |
| ENSG00000119508 | NR4A3 | -1.2472 | **0.0084** |
| ENSG00000120471 | TP53AIP1 | -1.3105 | **0.0263** |
| ENSG00000120738 | EGR1 | -1.2792 | **0.0142** |
| ENSG00000121335 | PRB2 | -1.4370 | **0.0122** |
| ENSG00000122574 | WIPF3 | -1.0673 | **0.0422** |
| ENSG00000122877 | EGR2 | -1.0160 | **0.0178** |
| ENSG00000123689 | G0S2 | -2.1178 | **0.0003** |
| ENSG00000124749 | COL21A1 | -1.2439 | **0.0464** |
| ENSG00000125740 | FOSB | -1.9539 | **0.0001** |
| ENSG00000131015 | ULBP2 | -1.5184 | **0.0021** |
| ENSG00000131721 | RHOXF2 | -1.1714 | **0.0366** |
| ENSG00000133937 | GSC | -1.5300 | **0.0191** |
| ENSG00000134551 | PRH2 | -1.3334 | **0.0159** |
| ENSG00000135111 | TBX3 | -1.9221 | **0.0051** |
| ENSG00000135248 | FAM71F1 | -1.2124 | **0.0308** |
| ENSG00000135702 | CHST5 | -1.5604 | **0.0070** |
| ENSG00000136694 | IL36A | -1.1144 | **0.0150** |
| ENSG00000138135 | CH25H | -1.6572 | **0.0043** |
| ENSG00000139209 | SLC38A4 | -1.6550 | **0.0039** |
| ENSG00000139287 | TPH2 | -1.1229 | **0.0382** |
| ENSG00000140254 | DUOXA1 | -1.1275 | **0.0182** |
| ENSG00000140527 | WDR93 | -1.3238 | **0.0096** |
| ENSG00000144481 | TRPM8 | -1.7174 | **0.0015** |
| ENSG00000149256 | TENM4 | -1.2345 | **0.0385** |
| ENSG00000151967 | SCHIP1 | -1.7957 | **0.0001** |
| ENSG00000153234 | NR4A2 | -1.4413 | **0.0002** |
| ENSG00000154479 | CCDC173 | -2.5295 | **0.0000** |
| ENSG00000154556 | SORBS2 | -1.5525 | **0.0013** |
| ENSG00000155011 | DKK2 | -1.5962 | **0.0010** |
| ENSG00000157613 | CREB3L1 | -2.4594 | **0.0003** |
| ENSG00000158050 | DUSP2 | -1.0087 | **0.0011** |
| ENSG00000159289 | GOLGA6A | -1.9467 | **0.0008** |
| ENSG00000160868 | CYP3A4 | -1.6154 | **0.0051** |
| ENSG00000162772 | ATF3 | -1.2461 | **0.0005** |
| ENSG00000163046 | ANKRD30BL | -1.7815 | **0.0022** |
| ENSG00000164729 | SLC35G3 | -1.1608 | **0.0445** |
| ENSG00000164761 | TNFRSF11B | -1.1854 | **0.0450** |
| ENSG00000164920 | OSR2 | -1.7158 | **0.0035** |
| ENSG00000165192 | ASB11 | -1.3132 | **0.0133** |
| ENSG00000165449 | SLC16A9 | -1.0577 | **0.0442** |
| ENSG00000167618 | LAIR2 | -1.0349 | **0.0011** |
| ENSG00000168135 | KCNJ4 | -1.9190 | **0.0009** |
| ENSG00000168348 | INSM2 | -1.2305 | **0.0311** |
| ENSG00000168490 | PHYHIP | -2.3328 | **0.0072** |
| ENSG00000169429 | CXCL8 | -1.2018 | **0.0051** |
| ENSG00000170608 | FOXA3 | -1.1271 | **0.0459** |
| ENSG00000171532 | NEUROD2 | -1.2421 | **0.0261** |
| ENSG00000172000 | ZNF556 | -1.1681 | **0.0306** |
| ENSG00000172752 | COL6A5 | -1.5800 | **0.0079** |
| ENSG00000173976 | RAX2 | -1.2316 | **0.0366** |
| ENSG00000174448 | STARD6 | -1.1327 | **0.0338** |
| ENSG00000174990 | CA5A | -1.2686 | **0.0294** |
| ENSG00000175514 | GPR152 | -1.4162 | **0.0003** |
| ENSG00000175592 | FOSL1 | -1.5081 | **0.0090** |
| ENSG00000176040 | TMPRSS7 | -1.7246 | **0.0021** |
| ENSG00000177464 | GPR4 | -1.4048 | **0.0068** |
| ENSG00000177606 | JUN | -1.1141 | **0.0000** |
| ENSG00000178021 | TSPYL6 | -1.1101 | **0.0381** |
| ENSG00000178821 | TMEM52 | -1.7289 | **0.0038** |
| ENSG00000179094 | AC129492.1 | -1.3512 | **0.0000** |
| ENSG00000179168 | GGN | -1.0676 | **0.0008** |
| ENSG00000179388 | EGR3 | -1.6323 | **0.0051** |
| ENSG00000179603 | GRM8 | -1.5781 | **0.0079** |
| ENSG00000179776 | CDH5 | -1.8358 | **0.0100** |
| ENSG00000180929 | GPR62 | -1.2301 | **0.0353** |
| ENSG00000181240 | SLC25A41 | -1.8605 | **0.0028** |
| ENSG00000181773 | GPR3 | -1.0360 | **0.0438** |
| ENSG00000182393 | IFNL1 | -1.0172 | **0.0293** |
| ENSG00000182667 | NTM | -1.2316 | **0.0318** |
| ENSG00000183160 | TMEM119 | -1.0358 | **0.0003** |
| ENSG00000183287 | CCBE1 | -2.1711 | **0.0004** |
| ENSG00000183549 | ACSM5 | -1.8211 | **0.0017** |
| ENSG00000183607 | GKN2 | -1.3665 | **0.0157** |
| ENSG00000183628 | DGCR6 | -1.1030 | **0.0010** |
| ENSG00000183695 | MRGPRX2 | -1.4570 | **0.0135** |
| ENSG00000183729 | NPBWR1 | -1.2162 | **0.0381** |
| ENSG00000184185 | KCNJ12 | -1.3353 | **0.0044** |
| ENSG00000185022 | MAFF | -1.0801 | **0.0003** |
| ENSG00000185264 | TEX33 | -1.3147 | **0.0239** |
| ENSG00000185467 | KPNA7 | -1.6778 | **0.0061** |
| ENSG00000185863 | TMEM210 | -2.0096 | **0.0010** |
| ENSG00000186417 | GLDN | -1.4904 | **0.0039** |
| ENSG00000187957 | DNER | -1.0792 | **0.0201** |
| ENSG00000188219 | POTEE | -1.0446 | **0.0101** |
| ENSG00000189120 | SP6 | -1.2064 | **0.0108** |
| ENSG00000197241 | SLC2A7 | -1.2999 | **0.0167** |
| ENSG00000198049 | AVPR1B | -1.3431 | **0.0182** |
| ENSG00000198729 | PPP1R14C | -1.1073 | **0.0037** |
| ENSG00000203972 | GLYATL3 | -2.1893 | **0.0047** |
| ENSG00000204099 | NEU4 | -1.1537 | **0.0025** |
| ENSG00000205710 | C17orf107 | -1.7865 | **0.0001** |
| ENSG00000214102 | WEE2 | -1.2622 | **0.0220** |
| ENSG00000214940 | NPIPA8 | -1.4327 | **0.0124** |
| ENSG00000243955 | GSTA1 | -1.1919 | **0.0345** |
| ENSG00000256797 | KLRF2 | -1.5000 | **0.0042** |
| ENSG00000260537 | AC012184.2 | -1.1821 | **0.0477** |
| ENSG00000267952 | AC008878.1 | -1.2375 | **0.0308** |
| ENSG00000275572 | GRIFIN | -1.0004 | **0.0253** |
| ENSG00000275895 | U2AF1L5 | -1.1902 | **0.0306** |
| ENSG00000277196 | AC007325.2 | -1.4521 | **0.0150** |
| ENSG00000283154 | IQCJ-SCHIP1 | -2.1221 | **0.0003** |
| ENSG00000286102 | AP000552.3 | -2.1255 | **0.0005** |

The bold text indicates significance.

**Table S4** Correlation analysis between differentially expressed lncRNAs and serum IgE.

| IgE | r | | | | *P* | | | |
| --- | --- | --- | --- | --- | --- | --- | --- | --- |
|  | a | b | c | d | a | b | c | d |
| Total IgE (kUA/L) | 0.1042 | -0.0533 | 0.0645 | -0.1912 | 0.6531 | 0.8186 | 0.7813 | 0.4063 |
| D1 (kUA/L) | -0.1999 | -0.2371 | 0.0791 | 0.1046 | 0.3851 | 0.3007 | 0.7332 | 0.6518 |
| D2 (kUA/L) | 0.1177 | 0.0417 | -0.0156 | 0.3331 | 0.6115 | 0.8575 | 0.9464 | 0.1401 |

'a' represents the *P* value for the comparison between VAS and relative expression of AC011524.2; 'b' represents the *P* value for the comparison between VAS and relative expression of AC121247.1; 'c' represents the *P* value for the comparison between VAS and relative expression of AL133371.3; 'd' represents the *P* value for the comparison between VAS and relative expression of AC106801.1. IgE, immunoglobulin E; D1, Dermatophagoides pteronyssinus; D2, Dermatophagoides farina.

**Table S5** Correlation analysis between VAS score and serum IgE.

| Inflammatory cytokine | VAS score | r | *P* |
| --- | --- | --- | --- |
| IgE | Total score | 0.1805 | 0.4338 |
|  | Nasal obstruction | -0.1168 | 0.6140 |
|  | Nasal pruritus | 0.1536 | 0.5062 |
|  | Sneeze | 0.3020 | 0.1833 |
|  | Runny nose | -0.1487 | 0.5201 |
|  | Itchy eyes | 0.3887 | 0.0816 |
|  | Tearing | -0.1977 | 0.3904 |
|  | Red eyes | 0.2326 | 0.3104 |
|  | Eye pain | 0.4054 | 0.0683 |
|  | Cough | -0.1934 | 0.4008 |
|  | Breath holding | 0.0557 | 0.8104 |
|  | Wheezing | 0.0740 | 0.7498 |
|  | Pressure sensation | 0.0980 | 0.6725 |
| D1 | Total score | -0.2544 | 0.2657 |
|  | Nasal obstruction | -0.2788 | 0.2209 |
|  | Nasal pruritus | -0.2315 | 0.3127 |
|  | Sneeze | 0.0438 | 0.8505 |
|  | Runny nose | 0.0492 | 0.8324 |
|  | Itchy eyes | -0.1456 | 0.5289 |
|  | Tearing | -0.4257 | 0.0543 |
|  | Red eyes | -0.1587 | 0.4921 |
|  | Eye pain | 0.2902 | 0.2019 |
|  | Cough | -0.1313 | 0.5704 |
|  | Breath holding | 0.1035 | 0.6551 |
|  | Wheezing | 0.3650 | 0.1037 |
|  | Pressure sensation | -0.2486 | 0.2771 |
| D2 | Total score | 0.1627 | 0.4809 |
|  | Nasal obstruction | -0.0294 | 0.8995 |
|  | Nasal pruritus | 0.1861 | 0.4194 |
|  | Sneeze | 0.4194 | 0.0584 |
|  | Runny nose | 0.1485 | 0.5205 |
|  | Itchy eyes | 0.1449 | 0.5307 |
|  | Tearing | -0.1044 | 0.6525 |
|  | Red eyes | -0.3370 | 0.1352 |
|  | Eye pain | -0.1028 | 0.6574 |
|  | Cough | 0.1243 | 0.5915 |
|  | Breath holding | -0.1517 | 0.5117 |
|  | Wheezing | -0.0486 | 0.8344 |
|  | Pressure sensation | -0.1458 | 0.5282 |

IgE, immunoglobulin E; D1, Dermatophagoides pteronyssinus; D2, Dermatophagoides farina.

**Table S6** Expression levels of serum inflammatory cytokines.

| Inflammatory cytokine (pg/mL) | HC | AR | *P* |
| --- | --- | --- | --- |
| CXCL5 | 1573.98±667.43 | 1463.29±708.82 | 0.6151 |
| FGF basic | 17.07±3.02 | 23.35±12.86 | **0.0446** |
| G-CSF | 15.58±9.63 | 25.32±27.27 | 0.1486 |
| GM-CSF | 3.91±4.51 | 2.97±1.71 | 0.3796 |
| IFN-γ | 1.82±2.70 | 2.15±2.65 | 0.6959 |
| IL-1β | 3.46±2.68 | 6.34±8.27 | 0.1550 |
| IL-2 | 12.58±8.53 | 14.50±8.71 | 0.4875 |
| IL-4 | 10.84±3.54 | 16.42±6.01 | **0.0011** |
| IL-5 | 3.53±0.82 | 3.28±1.13 | 0.4464 |
| IL-6 | 2.34±0.60 | 4.00±3.75 | 0.0638 |
| CXCL8 | 75.26±67.71 | 198.86±255.64 | **0.0482** |
| IL-10 | 1.96±0.67 | 2.05±1.08 | 0.7605 |
| IL-17 | 0.91±0.43 | 1.29±0.66 | **0.0364** |
| CCL2 | 184.34±64.26 | 209.65±66.92 | 0.2308 |
| CCL3 | 82.59±42.95 | 305.60±690.79 | **0.0440** |
| CCL4 | 123.68±70.89 | 34.68±152.3 | 0.0946 |
| CCL5 | 10123.44±5754.09 | 10992.96±6111.71 | 0.6471 |
| TNF-ɑ | 5.57±2.44 | 5.71±2.69 | 0.8628 |
| Tpo | 811.68±245.32 | 1082.05±661.99 | 0.1012 |
| VEGF | 83.81±53.91 | 82.09±49.60 | 0.9169 |

The data are expressed as the means ± standard deviations (SDs). The bold text indicates significance.

**Table S7** Correlation analysis between VAS score and inflammatory cytokines in the serum.

| Inflammatory cytokine in serum | VAS score | r | *P* |
| --- | --- | --- | --- |
| CXCL5 | Total score | -0.0704 | 0.6660 |
|  | Nasal obstruction | -0.0148 | 0.9276 |
|  | Nasal pruritus | -0.0857 | 0.5990 |
|  | Sneeze | -0.1966 | 0.2240 |
|  | Runny nose | -0.1134 | 0.4859 |
|  | Itchy eyes | -0.1397 | 0.3900 |
|  | Tearing | 0.0898 | 0.5815 |
|  | Red eyes | 0.1436 | 0.3768 |
|  | Eye pain | -0.0484 | 0.7670 |
|  | Cough | 0.2076 | 0.1988 |
|  | Breath holding | 0.0159 | 0.9226 |
|  | Wheezing | 0.1321 | 0.4163 |
|  | Pressure sensation | -0.2229 | 0.1668 |
| FGF basic | Total score | 0.2807 | 0.0793 |
|  | Nasal obstruction | 0.1865 | 0.2491 |
|  | Nasal pruritus | 0.3922 | **0.0123** |
|  | Sneeze | 0.3037 | 0.0567 |
|  | Runny nose | 0.2483 | 0.1224 |
|  | Itchy eyes | 0.2415 | 0.1333 |
|  | Tearing | 0.2478 | 0.1231 |
|  | Red eyes | 0.0336 | 0.8368 |
|  | Eye pain | 0.1639 | 0.3122 |
|  | Cough | 0.0009 | 0.9957 |
|  | Breath holding | 0.0235 | 0.8857 |
|  | Wheezing | -0.0968 | 0.5522 |
|  | Pressure sensation | 0.1245 | 0.4439 |
| G-CSF | Total score | 0.1969 | 0.2234 |
|  | Nasal obstruction | 0.1764 | 0.2763 |
|  | Nasal pruritus | 0.2052 | 0.2039 |
|  | Sneeze | 0.1161 | 0.4757 |
|  | Runny nose | 0.2016 | 0.2123 |
|  | Itchy eyes | 0.1134 | 0.4861 |
|  | Tearing | 0.2131 | 0.1867 |
|  | Red eyes | 0.0625 | 0.7018 |
|  | Eye pain | 0.3055 | 0.0552 |
|  | Cough | 0.1237 | 0.4469 |
|  | Breath holding | 0.3422 | 0.0307 |
|  | Wheezing | -0.1111 | 0.4950 |
|  | Pressure sensation | -0.1116 | 0.4929 |
| GM-CSF | Total score | -0.1767 | 0.2753 |
|  | Nasal obstruction | -0.1512 | 0.3517 |
|  | Nasal pruritus | -0.1562 | 0.3359 |
|  | Sneeze | -0.2158 | 0.1811 |
|  | Runny nose | -0.1323 | 0.4157 |
|  | Itchy eyes | -0.2277 | 0.1576 |
|  | Tearing | -0.1120 | 0.4912 |
|  | Red eyes | -0.0470 | 0.7735 |
|  | Eye pain | -0.0671 | 0.6809 |
|  | Cough | -0.0884 | 0.5874 |
|  | Breath holding | 0.0943 | 0.5628 |
|  | Wheezing | 0.0849 | 0.6025 |
|  | Pressure sensation | 0.0175 | 0.9148 |
| IFN-ץ | Total score | 0.0401 | 0.8059 |
|  | Nasal obstruction | 0.0269 | 0.8690 |
|  | Nasal pruritus | 0.0726 | 0.6563 |
|  | Sneeze | 0.0645 | 0.6924 |
|  | Runny nose | -0.0182 | 0.9114 |
|  | Itchy eyes | 0.0069 | 0.9664 |
|  | Tearing | 0.1045 | 0.5211 |
|  | Red eyes | -0.0419 | 0.7972 |
|  | Eye pain | -0.0211 | 0.8970 |
|  | Cough | 0.0367 | 0.8223 |
|  | Breath holding | 0.1407 | 0.3865 |
|  | Wheezing | -0.1573 | 0.3322 |
|  | Pressure sensation | 0.1067 | 0.5122 |
| IL-1 β | Total score | 0.1916 | 0.2362 |
|  | Nasal obstruction | 0.2173 | 0.1780 |
|  | Nasal pruritus | 0.2981 | **0.0617** |
|  | Sneeze | 0.1889 | 0.2430 |
|  | Runny nose | 0.0977 | 0.5485 |
|  | Itchy eyes | 0.2093 | 0.1949 |
|  | Tearing | 0.1519 | 0.3494 |
|  | Red eyes | 0.0474 | 0.7713 |
|  | Eye pain | -0.0134 | 0.9344 |
|  | Cough | 0.0011 | 0.9948 |
|  | Breath holding | -0.1224 | 0.4518 |
|  | Wheezing | -0.1398 | 0.3897 |
|  | Pressure sensation | -0.0665 | 0.6834 |
| IL-2 | Total score | 0.1101 | 0.4990 |
|  | Nasal obstruction | 0.1547 | 0.3404 |
|  | Nasal pruritus | 0.1202 | 0.4599 |
|  | Sneeze | 0.0269 | 0.8691 |
|  | Runny nose | 0.0575 | 0.7247 |
|  | Itchy eyes | 0.0095 | 0.9534 |
|  | Tearing | 0.1624 | 0.3168 |
|  | Red eyes | 0.1828 | 0.2590 |
|  | Eye pain | -0.1525 | 0.3475 |
|  | Cough | 0.0735 | 0.6521 |
|  | Breath holding | 0.4338 | **0.0052** |
|  | Wheezing | -0.0466 | 0.7751 |
|  | Pressure sensation | -0.0517 | 0.7513 |
| IL-4 | Total score | 0.4912 | **0.0013** |
|  | Nasal obstruction | 0.4128 | **0.0081** |
|  | Nasal pruritus | 0.4731 | **0.0020** |
|  | Sneeze | 0.3966 | **0.0113** |
|  | Runny nose | 0.4341 | **0.0051** |
|  | Itchy eyes | 0.4609 | **0.0028** |
|  | Tearing | 0.3689 | **0.0192** |
|  | Red eyes | 0.3796 | **0.0157** |
|  | Eye pain | 0.4390 | **0.0046** |
|  | Cough | 0.1629 | 0.3152 |
|  | Breath holding | 0.2105 | 0.1922 |
|  | Wheezing | -0.1128 | 0.4884 |
|  | Pressure sensation | 0.0501 | 0.7587 |
| IL-5 | Total score | -0.1592 | 0.3264 |
|  | Nasal obstruction | -0.1490 | 0.3588 |
|  | Nasal pruritus | -0.0948 | 0.5607 |
|  | Sneeze | -0.1013 | 0.5340 |
|  | Runny nose | -0.0484 | 0.7668 |
|  | Itchy eyes | -0.1346 | 0.4075 |
|  | Tearing | -0.1201 | 0.4605 |
|  | Red eyes | -0.3908 | **0.0126** |
|  | Eye pain | -0.3720 | 0.0181 |
|  | Cough | -0.1075 | 0.5089 |
|  | Breath holding | -0.0567 | 0.7282 |
|  | Wheezing | 0.0941 | 0.5636 |
|  | Pressure sensation | 0.1077 | 0.5085 |
| IL-6 | Total score | 0.2523 | 0.1162 |
|  | Nasal obstruction | 0.1724 | 0.2874 |
|  | Nasal pruritus | 0.3149 | **0.0478** |
|  | Sneeze | 0.2985 | 0.0613 |
|  | Runny nose | 0.2708 | 0.0910 |
|  | Itchy eyes | 0.2858 | 0.0738 |
|  | Tearing | 0.0810 | 0.6192 |
|  | Red eyes | -0.0568 | 0.7280 |
|  | Eye pain | 0.3638 | **0.0210** |
|  | Cough | 0.0535 | 0.7428 |
|  | Breath holding | -0.0233 | 0.8865 |
|  | Wheezing | -0.1108 | 0.4961 |
|  | Pressure sensation | -0.0415 | 0.7994 |
| CXCL8 | Total score | 0.2505 | 0.1190 |
|  | Nasal obstruction | 0.2851 | 0.0746 |
|  | Nasal pruritus | 0.3725 | **0.0179** |
|  | Sneeze | 0.2548 | 0.1126 |
|  | Runny nose | 0.1767 | 0.2755 |
|  | Itchy eyes | 0.2715 | 0.0902 |
|  | Tearing | 0.1570 | 0.3334 |
|  | Red eyes | -0.0470 | 0.7735 |
|  | Eye pain | 0.0765 | 0.6388 |
|  | Cough | -0.0080 | 0.9608 |
|  | Breath holding | -0.0659 | 0.6863 |
|  | Wheezing | -0.0804 | 0.6219 |
|  | Pressure sensation | -0.1107 | 0.4966 |
| IL-10 | Total score | 0.0724 | 0.6569 |
|  | Nasal obstruction | 0.0327 | 0.8412 |
|  | Nasal pruritus | 0.0413 | 0.8003 |
|  | Sneeze | 0.1783 | 0.2711 |
|  | Runny nose | 0.0006 | 0.9969 |
|  | Itchy eyes | 0.2232 | 0.1663 |
|  | Tearing | -0.1607 | 0.3217 |
|  | Red eyes | 0.0428 | 0.7930 |
|  | Eye pain | 0.4525 | **0.0034** |
|  | Cough | -0.0491 | 0.7636 |
|  | Breath holding | -0.0611 | 0.7079 |
|  | Wheezing | -0.0946 | 0.5617 |
|  | Pressure sensation | -0.1015 | 0.5334 |
| IL-17 | Total score | 0.3127 | **0.0495** |
|  | Nasal obstruction | 0.2903 | 0.0692 |
|  | Nasal pruritus | 0.2420 | 0.1324 |
|  | Sneeze | 0.2011 | 0.2133 |
|  | Runny nose | 0.2853 | 0.0743 |
|  | Itchy eyes | 0.2286 | 0.1559 |
|  | Tearing | 0.3949 | **0.0117** |
|  | Red eyes | 0.3244 | **0.0411** |
|  | Eye pain | 0.1604 | 0.3229 |
|  | Cough | 0.0195 | 0.9050 |
|  | Breath holding | 0.0838 | 0.6070 |
|  | Wheezing | 0.0075 | 0.9633 |
|  | Pressure sensation | 0.0351 | 0.8299 |
| CCL2 | Total score | 0.2227 | 0.1672 |
|  | Nasal obstruction | 0.1955 | 0.2266 |
|  | Nasal pruritus | 0.2204 | 0.1717 |
|  | Sneeze | 0.2181 | 0.1763 |
|  | Runny nose | 0.2193 | 0.1740 |
|  | Itchy eyes | 0.1588 | 0.3278 |
|  | Tearing | 0.1716 | 0.2896 |
|  | Red eyes | 0.1217 | 0.4545 |
|  | Eye pain | -0.1790 | 0.2691 |
|  | Cough | 0.1216 | 0.4547 |
|  | Breath holding | 0.3770 | **0.0165** |
|  | Wheezing | -0.1366 | 0.4005 |
|  | Pressure sensation | 0.0968 | 0.5523 |
| CCL3 | Total score | 0.1746 | 0.2812 |
|  | Nasal obstruction | 0.1724 | 0.2873 |
|  | Nasal pruritus | 0.3141 | **0.0484** |
|  | Sneeze | 0.2442 | 0.1289 |
|  | Runny nose | 0.0963 | 0.5545 |
|  | Itchy eyes | 0.2528 | 0.1155 |
|  | Tearing | 0.0243 | 0.8817 |
|  | Red eyes | -0.1033 | 0.5260 |
|  | Eye pain | -0.0422 | 0.7958 |
|  | Cough | 0.0507 | 0.7561 |
|  | Breath holding | -0.0676 | 0.6785 |
|  | Wheezing | -0.0644 | 0.6930 |
|  | Pressure sensation | -0.0577 | 0.7237 |
| CCL4 | Total score | 0.2791 | 0.0812 |
|  | Nasal obstruction | 0.2250 | 0.1628 |
|  | Nasal pruritus | 0.3596 | **0.0227** |
|  | Sneeze | 0.3613 | 0.0220 |
|  | Runny nose | 0.2290 | 0.1551 |
|  | Itchy eyes | 0.3140 | 0.0485 |
|  | Tearing | 0.1902 | 0.2399 |
|  | Red eyes | -0.0790 | 0.6281 |
|  | Eye pain | -0.1098 | 0.5002 |
|  | Cough | 0.1309 | 0.4209 |
|  | Breath holding | -0.0132 | 0.9358 |
|  | Wheezing | -0.0770 | 0.6366 |
|  | Pressure sensation | -0.0115 | 0.9441 |
| CCL5 | Total score | 0.0901 | 0.5802 |
|  | Nasal obstruction | 0.0586 | 0.7196 |
|  | Nasal pruritus | 0.0663 | 0.6843 |
|  | Sneeze | 0.0961 | 0.5554 |
|  | Runny nose | 0.1890 | 0.2429 |
|  | Itchy eyes | 0.0040 | 0.9807 |
|  | Tearing | 0.0534 | 0.7435 |
|  | Red eyes | -0.0124 | 0.9395 |
|  | Eye pain | -0.0918 | 0.5730 |
|  | Cough | 0.1908 | 0.2381 |
|  | Breath holding | -0.0496 | 0.7612 |
|  | Wheezing | -0.1904 | 0.2394 |
|  | Pressure sensation | 0.1632 | 0.3143 |
| TNF-ɑ | Total score | -0.0292 | 0.8579 |
|  | Nasal obstruction | -0.0725 | 0.6567 |
|  | Nasal pruritus | 0.0466 | 0.7755 |
|  | Sneeze | 0.0517 | 0.7514 |
|  | Runny nose | -0.0539 | 0.7414 |
|  | Itchy eyes | -0.0314 | 0.8476 |
|  | Tearing | -0.0248 | 0.8792 |
|  | Red eyes | -0.1212 | 0.4561 |
|  | Eye pain | 0.1104 | 0.4979 |
|  | Cough | -0.1756 | 0.2784 |
|  | Breath holding | 0.1082 | 0.5063 |
|  | Wheezing | -0.0946 | 0.5616 |
|  | Pressure sensation | -0.0167 | 0.9188 |
| Tpo | Total score | 0.2669 | 0.0960 |
|  | Nasal obstruction | 0.2360 | 0.1426 |
|  | Nasal pruritus | 0.2491 | 0.1212 |
|  | Sneeze | 0.3054 | 0.0553 |
|  | Runny nose | 0.2645 | 0.0991 |
|  | Itchy eyes | 0.1978 | 0.2211 |
|  | Tearing | 0.3021 | 0.0581 |
|  | Red eyes | -0.0577 | 0.7237 |
|  | Eye pain | -0.0176 | 0.9141 |
|  | Cough | -0.0213 | 0.8963 |
|  | Breath holding | 0.0608 | 0.7093 |
|  | Wheezing | -0.1246 | 0.4438 |
|  | Pressure sensation | 0.1405 | 0.3874 |
| VEGF | Total score | 0.0249 | 0.8786 |
|  | Nasal obstruction | -0.0667 | 0.6827 |
|  | Nasal pruritus | -0.0028 | 0.9863 |
|  | Sneeze | 0.0171 | 0.9166 |
|  | Runny nose | 0.0792 | 0.6270 |
|  | Itchy eyes | 0.0224 | 0.8907 |
|  | Tearing | 0.0925 | 0.5704 |
|  | Red eyes | 0.0437 | 0.7891 |
|  | Eye pain | -0.0489 | 0.7645 |
|  | Cough | -0.0535 | 0.7431 |
|  | Breath holding | 0.1136 | 0.4853 |
|  | Wheezing | -0.1360 | 0.4026 |
|  | Pressure sensation | 0.0635 | 0.6972 |

The bold text indicates significance.

**Table S8** Correlation analysis between inflammatory factors in the serum.

| Inflammatory cytokine in serum | Inflammatory cytokine in serum | r | *P* |
| --- | --- | --- | --- |
| CXCL5 | FGF basic | -0.3165 | **0.0466** |
|  | G-CSF | -0.1734 | 0.2846 |
|  | GM-CSF | 0.1219 | 0.4538 |
|  | IFN-γ | -0.0262 | 0.8727 |
|  | IL-1β | -0.0624 | 0.7023 |
|  | IL-2 | 0.0827 | 0.6120 |
|  | IL-4 | -0.0937 | 0.5653 |
|  | IL-5 | 0.0729 | 0.6547 |
|  | IL-6 | -0.3075 | 0.0536 |
|  | CXCL8 | -0.1996 | 0.2168 |
|  | IL-10 | -0.2971 | 0.0627 |
|  | IL-17 | -0.2140 | 0.1849 |
|  | CCL2 | 0.0380 | 0.8158 |
|  | CCL3 | -0.1860 | 0.2505 |
|  | CCL4 | -0.1071 | 0.5106 |
|  | CCL5 | -0.2517 | 0.1172 |
|  | TNF-ɑ | -0.2033 | 0.2083 |
|  | Tpo | -0.0881 | 0.5887 |
|  | VEGF | -0.1109 | 0.4957 |
| FGF basic | G-CSF | 0.5076 | **0.0008** |
|  | GM-CSF | 0.0042 | 0.9795 |
|  | IFN-γ | 0.1738 | 0.2836 |
|  | IL-1β | 0.5694 | **0.0001** |
|  | IL-2 | 0.1453 | 0.3710 |
|  | IL-4 | 0.5930 | **0.0001** |
|  | IL-5 | 0.0764 | 0.6395 |
|  | IL-6 | 0.8686 | **<0.0001** |
|  | CXCL8 | 0.7850 | **<0.0001** |
|  | IL-10 | 0.2069 | 0.2002 |
|  | IL-17 | 0.0433 | 0.7906 |
|  | CCL2 | 0.1845 | 0.2543 |
|  | CCL3 | 0.7014 | **<0.0001** |
|  | CCL4 | 0.6294 | **<0.0001** |
|  | CCL5 | -0.0097 | 0.9524 |
|  | TNF-ɑ | 0.5935 | **<0.0001** |
|  | Tpo | -0.0055 | 0.9730 |
|  | VEGF | 0.1954 | 0.2268 |
| G-CSF | GM-CSF | -0.0495 | 0.7615 |
|  | IFN-γ | 0.3013 | 0.0589 |
|  | IL-1β | -0.0475 | 0.7708 |
|  | IL-2 | 0.1606 | 0.3223 |
|  | IL-4 | 0.5290 | **0.0004** |
|  | IL-5 | -0.0003 | 0.9987 |
|  | IL-6 | 0.6062 | **<0.0001** |
|  | CXCL8 | 0.2781 | 0.0822 |
|  | IL-10 | 0.2698 | 0.0923 |
|  | IL-17 | 0.0623 | 0.7026 |
|  | CCL2 | 0.0970 | 0.5517 |
|  | CCL3 | 0.0914 | 0.5747 |
|  | CCL4 | 0.1809 | 0.2640 |
|  | CCL5 | -0.0477 | 0.7701 |
|  | TNF-ɑ | 0.4411 | **0.0044** |
|  | Tpo | -0.0150 | 0.9269 |
|  | VEGF | 0.2360 | 0.1426 |
| GM-CSF | IFN-γ | 0.3116 | 0.0503 |
|  | IL-1β | -0.0952 | 0.5588 |
|  | IL-2 | 0.1158 | 0.4767 |
|  | IL-4 | -0.0842 | 0.6054 |
|  | IL-5 | 0.1382 | 0.3950 |
|  | IL-6 | 0.0116 | 0.9433 |
|  | CXCL8 | -0.0596 | 0.7150 |
|  | IL-10 | -0.0094 | 0.9542 |
|  | IL-17 | -0.2469 | 0.1245 |
|  | CCL2 | 0.2579 | 0.1082 |
|  | CCL3 | -0.0046 | 0.9775 |
|  | CCL4 | 0.1910 | 0.2379 |
|  | CCL5 | -0.2983 | 0.0615 |
|  | TNF-ɑ | 0.5081 | **0.0008** |
|  | Tpo | -0.0316 | 0.8465 |
|  | VEGF | -0.0425 | 0.7944 |
| IFN-γ | IL-1β | 0.0883 | 0.5879 |
|  | IL-2 | -0.1519 | 0.3495 |
|  | IL-4 | -0.1504 | 0.3542 |
|  | IL-5 | 0.2070 | 0.1999 |
|  | IL-6 | 0.0640 | 0.6948 |
|  | CXCL8 | 0.1818 | 0.2615 |
|  | IL-10 | -0.0768 | 0.6377 |
|  | IL-17 | -0.3325 | **0.0360** |
|  | CCL2 | -0.0698 | 0.6688 |
|  | CCL3 | -0.0258 | 0.8743 |
|  | CCL4 | 0.0022 | 0.9893 |
|  | CCL5 | -0.0243 | 0.8817 |
|  | TNF-ɑ | 0.2882 | 0.0713 |
|  | Tpo | -0.2178 | 0.1770 |
|  | VEGF | -0.2338 | 0.1465 |
| IL-1β | IL-2 | 0.0287 | 0.8607 |
|  | IL-4 | 0.2657 | 0.0976 |
|  | IL-5 | 0.0308 | 0.8501 |
|  | IL-6 | 0.4346 | **0.0051** |
|  | CXCL8 | 0.8885 | **<0.0001** |
|  | IL-10 | -0.0943 | 0.5626 |
|  | IL-17 | -0.0588 | 0.7184 |
|  | CCL2 | 0.0370 | 0.8208 |
|  | CCL3 | 0.8631 | **<0.0001** |
|  | CCL4 | 0.5781 | **0.0001** |
|  | CCL5 | 0.1480 | 0.3619 |
|  | TNF-ɑ | 0.2068 | 0.2005 |
|  | Tpo | -0.0220 | 0.8929 |
|  | VEGF | -0.1423 | 0.3809 |
| IL-2 | IL-4 | 0.4687 | **0.0023** |
|  | IL-5 | -0.1065 | 0.5132 |
|  | IL-6 | 0.0954 | 0.5581 |
|  | CXCL8 | 0.0909 | 0.5772 |
|  | IL-10 | 0.2552 | 0.1120 |
|  | IL-17 | 0.3536 | **0.0252** |
|  | CCL2 | 0.8154 | **<0.0001** |
|  | CCL3 | 0.1688 | 0.2978 |
|  | CCL4 | 0.2931 | 0.0664 |
|  | CCL5 | 0.1005 | 0.5374 |
|  | TNF-ɑ | 0.3272 | **0.0393** |
|  | Tpo | 0.1821 | 0.2608 |
|  | VEGF | 0.5869 | **0.0001** |
| IL-4 | IL-5 | -0.2496 | 0.1203 |
|  | IL-6 | 0.7198 | **0.0000** |
|  | CXCL8 | 0.4292 | **0.0057** |
|  | IL-10 | 0.4993 | **0.0010** |
|  | IL-17 | 0.4163 | **0.0075** |
|  | CCL2 | 0.4826 | **0.0016** |
|  | CCL3 | 0.3817 | **0.0151** |
|  | CCL4 | 0.5556 | **0.0002** |
|  | CCL5 | -0.0432 | 0.7912 |
|  | TNF-ɑ | 0.4482 | **0.0037** |
|  | Tpo | 0.2636 | 0.1002 |
|  | VEGF | 0.4492 | **0.0036** |
| IL-5 | IL-6 | -0.0120 | 0.9413 |
|  | CXCL8 | 0.1391 | 0.3920 |
|  | IL-10 | -0.3212 | **0.0433** |
|  | IL-17 | -0.1922 | 0.2348 |
|  | CCL2 | 0.0148 | 0.9278 |
|  | CCL3 | 0.0747 | 0.6470 |
|  | CCL4 | 0.0615 | 0.7062 |
|  | CCL5 | -0.0889 | 0.5854 |
|  | TNF-ɑ | 0.1117 | 0.4925 |
|  | Tpo | -0.0753 | 0.6440 |
|  | VEGF | 0.0430 | 0.7920 |
| IL-6 | CXCL8 | 0.6744 | **<0.0001** |
|  | IL-10 | 0.4412 | **0.0044** |
|  | IL-17 | 0.0150 | 0.9268 |
|  | CCL2 | 0.2003 | 0.2152 |
|  | CCL3 | 0.6186 | **<0.0001** |
|  | CCL4 | 0.5926 | **0.0001** |
|  | CCL5 | -0.0896 | 0.5825 |
|  | TNF-ɑ | 0.5837 | **0.0001** |
|  | Tpo | 0.0924 | 0.5707 |
|  | VEGF | 0.1579 | 0.3305 |
| CXCL8 | IL-10 | 0.0748 | 0.6464 |
|  | IL-17 | -0.0784 | 0.6305 |
|  | CCL2 | 0.0940 | 0.5638 |
|  | CCL3 | 0.8580 | **<0.0001** |
|  | CCL4 | 0.6541 | **<0.0001** |
|  | CCL5 | 0.0889 | 0.5853 |
|  | TNF-ɑ | 0.4295 | **0.0057** |
|  | Tpo | -0.0383 | 0.8146 |
|  | VEGF | -0.0258 | 0.8745 |
| IL-10 | IL-17 | 0.1261 | 0.4381 |
|  | CCL2 | 0.2051 | 0.2042 |
|  | CCL3 | 0.0316 | 0.8464 |
|  | CCL4 | 0.0946 | 0.5615 |
|  | CCL5 | -0.2071 | 0.1998 |
|  | TNF-ɑ | 0.2968 | 0.0629 |
|  | Tpo | 0.0998 | 0.5401 |
|  | VEGF | 0.2555 | 0.1115 |
| IL-17 | CCL2 | 0.2860 | 0.0736 |
|  | CCL3 | -0.0685 | 0.6744 |
|  | CCL4 | 0.0108 | 0.9473 |
|  | CCL5 | 0.0931 | 0.5678 |
|  | TNF-ɑ | 0.0274 | 0.8668 |
|  | Tpo | 0.2108 | 0.1916 |
|  | VEGF | 0.4295 | **0.0057** |
| CCL2 | CCL3 | 0.2191 | 0.1743 |
|  | CCL4 | 0.4224 | **0.0066** |
|  | CCL5 | 0.2004 | 0.2151 |
|  | TNF-ɑ | 0.3962 | **0.0114** |
|  | Tpo | 0.3601 | **0.0225** |
|  | VEGF | 0.4937 | **0.0012** |
| CCL3 | CCL4 | 0.7580 | **<0.0001** |
|  | CCL5 | 0.1021 | 0.5308 |
|  | TNF-ɑ | 0.3564 | **0.0240** |
|  | Tpo | -0.0255 | 0.8761 |
|  | VEGF | -0.0281 | 0.8634 |
| CCL4 | CCL5 | 0.0188 | 0.9084 |
|  | TNF-ɑ | 0.4614 | **0.0027** |
|  | Tpo | 0.1430 | 0.3786 |
|  | VEGF | 0.1130 | 0.4874 |
| CCL5 | TNF-ɑ | -0.0675 | 0.6792 |
|  | Tpo | 0.0988 | 0.5442 |
|  | VEGF | -0.0156 | 0.9239 |
| TNF-ɑ | Tpo | 0.0145 | 0.9294 |
|  | VEGF | 0.2817 | 0.0782 |
| Tpo | VEGF | 0.4628 | 0.0026 |

The bold text indicates significance.

**Table S9** Expression levels of inflammatory cytokines in nasal secretions.

| Inflammatory cytokine (pg/mL) | HC | AR | *P* |
| --- | --- | --- | --- |
| CXCL5 | 1281.96±2652.11 | 1797.09±1664.48 | 0.3849 |
| FGF basic | 300.40±223.56 | 198.00±21.55 | 0.0638 |
| G-CSF | 685.77±548.39 | 1785.26±1737.35 | **0.0059** |
| GM-CSF | 8.74±1.64 | 8.85±2.08 | 0.7927 |
| IFN-γ | 3.23±0.85 | 5.96±3.94 | **0.0019** |
| IL-1β/IL-1F2 | 29.08±25.43 | 479.16±1113.32 | 0.0698 |
| IL-2 | 26.09±5.06 | 29.47±5.28 | 0.0584 |
| IL-4 | 44.11±4.68 | 57.00±21.22 | **0.0069** |
| IL-5 | 6.56±7.67 | 4.88±6.28 | 0.5599 |
| IL-6 | 12.97±9.47 | 41.26±53.09 | **0.0176** |
| CXCL8/IL-8 | 2276.02±1525.95 | 2569.74±1453.52 | 0.3141 |
| IL-10 | 2.43±0.74 | 2.85±0.94 | 0.1580 |
| IL-17 | 2.29±1.79 | 2.55±2.12 | 0.5173 |
| CCL2/MCP-1 | 77.07±67.31 | 94.72±76.45 | 0.3557 |
| CCL3/MIP-1ɑ | 320.09±37.51 | 427.50±414.65 | 0.2388 |
| CCL4/MIP-1β | 82.68±38.61 | 131.87±165.17 | 0.1659 |
| CCL5/RANTES | 69.06±49.44 | 111.11±115.63 | 0.1152 |
| TNF-ɑ | 10.61±4.82 | 20.96±18.28 | **0.0121** |
| Tpo | 158.88±19.18 | 178.30±35.82 | 0.0563 |
| VEGF | 226.15±93.74 | 289.00±203.46 | 0.1896 |

The data are expressed as the means ± standard deviations (SDs). The bold text indicates significance.

**Table S10** Correlation analysis between VAS score and inflammatory cytokines in nasal secretions.

| Inflammatory cytokine in serum | VAS score | r | *P* |
| --- | --- | --- | --- |
| CXCL5 | Total score | 0.1129 | 0.4879 |
|  | Nasal obstruction | 0.1002 | 0.5386 |
|  | Nasal pruritus | 0.0825 | 0.6129 |
|  | Sneeze | 0.0469 | 0.7737 |
|  | Runny nose | 0.1181 | 0.4681 |
|  | Itchy eyes | 0.0887 | 0.5864 |
|  | Tearing | 0.1462 | 0.3681 |
|  | Red eyes | 0.1118 | 0.4922 |
|  | Eye pain | 0.1049 | 0.5194 |
|  | Cough | -0.0041 | 0.9800 |
|  | Breath holding | -0.0106 | 0.9483 |
|  | Wheezing | -0.0674 | 0.6795 |
|  | Pressure sensation | 0.0531 | 0.7448 |
| FGF basic | Total score | -0.3204 | **0.0439** |
|  | Nasal obstruction | -0.3100 | 0.0516 |
|  | Nasal pruritus | -0.2919 | 0.0676 |
|  | Sneeze | -0.3118 | 0.0502 |
|  | Runny nose | -0.3451 | **0.0292** |
|  | Itchy eyes | -0.2870 | 0.0726 |
|  | Tearing | -0.1815 | 0.2624 |
|  | Red eyes | -0.0639 | 0.6955 |
|  | Eye pain | 0.0256 | 0.8756 |
|  | Cough | -0.1807 | 0.2645 |
|  | Breath holding | -0.1238 | 0.4465 |
|  | Wheezing | -0.0759 | 0.6417 |
|  | Pressure sensation | -0.1441 | 0.3751 |
| G-CSF | Total score | 0.2827 | 0.0771 |
|  | Nasal obstruction | 0.4550 | **0.0032** |
|  | Nasal pruritus | 0.2768 | 0.0838 |
|  | Sneeze | 0.0404 | 0.8044 |
|  | Runny nose | 0.2703 | 0.0916 |
|  | Itchy eyes | 0.0960 | 0.5557 |
|  | Tearing | 0.3329 | **0.0358** |
|  | Red eyes | 0.1549 | 0.3398 |
|  | Eye pain | 0.1141 | 0.4833 |
|  | Cough | 0.2141 | 0.1847 |
|  | Breath holding | 0.2030 | 0.2090 |
|  | Wheezing | 0.1799 | 0.2667 |
|  | Pressure sensation | -0.1741 | 0.2826 |
| GM-CSF | Total score | -0.0261 | 0.8732 |
|  | Nasal obstruction | 0.0769 | 0.6374 |
|  | Nasal pruritus | -0.0476 | 0.7707 |
|  | Sneeze | -0.2402 | 0.1354 |
|  | Runny nose | 0.1309 | 0.4207 |
|  | Itchy eyes | -0.1839 | 0.2560 |
|  | Tearing | 0.0047 | 0.9770 |
|  | Red eyes | 0.0901 | 0.5805 |
|  | Eye pain | -0.0741 | 0.6495 |
|  | Cough | 0.0792 | 0.6269 |
|  | Breath holding | 0.2067 | 0.2008 |
|  | Wheezing | 0.0231 | 0.8875 |
|  | Pressure sensation | 0.0209 | 0.8981 |
| IFN-ץ | Total score | 0.3224 | **0.0425** |
|  | Nasal obstruction | 0.5025 | **0.0010** |
|  | Nasal pruritus | 0.3264 | **0.0398** |
|  | Sneeze | 0.0381 | 0.8155 |
|  | Runny nose | 0.2546 | 0.1129 |
|  | Itchy eyes | 0.1001 | 0.5390 |
|  | Tearing | 0.4313 | **0.0055** |
|  | Red eyes | 0.2612 | 0.1035 |
|  | Eye pain | 0.1590 | 0.3271 |
|  | Cough | 0.2365 | 0.1417 |
|  | Breath holding | 0.2972 | 0.0625 |
|  | Wheezing | 0.2278 | 0.1575 |
|  | Pressure sensation | -0.1603 | 0.3230 |
| IL-1 β | Total score | 0.1588 | 0.3278 |
|  | Nasal obstruction | 0.3105 | 0.0512 |
|  | Nasal pruritus | 0.2029 | 0.2093 |
|  | Sneeze | -0.0618 | 0.7050 |
|  | Runny nose | 0.0596 | 0.7148 |
|  | Itchy eyes | -0.0375 | 0.8182 |
|  | Tearing | 0.2555 | 0.1115 |
|  | Red eyes | 0.2641 | 0.0996 |
|  | Eye pain | 0.1427 | 0.3797 |
|  | Cough | 0.0064 | 0.9687 |
|  | Breath holding | 0.4341 | **0.0051** |
|  | Wheezing | 0.4710 | **0.0022** |
|  | Pressure sensation | -0.0777 | 0.6337 |
| IL-2 | Total score | 0.2817 | 0.0782 |
|  | Nasal obstruction | 0.3468 | **0.0283** |
|  | Nasal pruritus | 0.2831 | 0.0767 |
|  | Sneeze | 0.2144 | 0.1839 |
|  | Runny nose | 0.2570 | 0.1094 |
|  | Itchy eyes | 0.2818 | 0.0782 |
|  | Tearing | 0.1017 | 0.5323 |
|  | Red eyes | 0.0975 | 0.5493 |
|  | Eye pain | 0.1374 | 0.3980 |
|  | Cough | 0.1441 | 0.3752 |
|  | Breath holding | 0.0858 | 0.5987 |
|  | Wheezing | 0.1448 | 0.3726 |
|  | Pressure sensation | -0.0347 | 0.8316 |
| IL-4 | Total score | 0.2795 | 0.0807 |
|  | Nasal obstruction | 0.4492 | **0.0036** |
|  | Nasal pruritus | 0.2726 | 0.0887 |
|  | Sneeze | -0.0047 | 0.9772 |
|  | Runny nose | 0.2590 | 0.1065 |
|  | Itchy eyes | 0.0774 | 0.6349 |
|  | Tearing | 0.3744 | **0.0173** |
|  | Red eyes | 0.2241 | 0.1646 |
|  | Eye pain | 0.1296 | 0.4253 |
|  | Cough | 0.2243 | 0.1641 |
|  | Breath holding | 0.2411 | 0.1339 |
|  | Wheezing | 0.1916 | 0.2362 |
|  | Pressure sensation | -0.1479 | 0.3624 |
| IL-5 | Total score | -0.0865 | 0.5957 |
|  | Nasal obstruction | -0.0893 | 0.5836 |
|  | Nasal pruritus | -0.0645 | 0.6925 |
|  | Sneeze | 0.0101 | 0.9507 |
|  | Runny nose | -0.2441 | 0.1290 |
|  | Itchy eyes | 0.0366 | 0.8224 |
|  | Tearing | -0.0783 | 0.6311 |
|  | Red eyes | 0.0383 | 0.8147 |
|  | Eye pain | 0.0593 | 0.7161 |
|  | Cough | -0.1541 | 0.3424 |
|  | Breath holding | -0.1537 | 0.3437 |
|  | Wheezing | 0.1626 | 0.3162 |
|  | Pressure sensation | -0.1156 | 0.4774 |
| IL-6 | Total score | 0.1993 | 0.2175 |
|  | Nasal obstruction | 0.3516 | **0.0261** |
|  | Nasal pruritus | 0.2495 | 0.1206 |
|  | Sneeze | 0.0791 | 0.6275 |
|  | Runny nose | 0.0947 | 0.5611 |
|  | Itchy eyes | 0.0473 | 0.7718 |
|  | Tearing | 0.3032 | 0.0572 |
|  | Red eyes | 0.0458 | 0.7792 |
|  | Eye pain | 0.0122 | 0.9407 |
|  | Cough | 0.0281 | 0.8635 |
|  | Breath holding | 0.2140 | 0.1848 |
|  | Wheezing | 0.2531 | 0.1151 |
|  | Pressure sensation | -0.1274 | 0.4334 |
| CXCL8 | Total score | 0.0503 | 0.7581 |
|  | Nasal obstruction | 0.1332 | 0.4127 |
|  | Nasal pruritus | 0.0600 | 0.7129 |
|  | Sneeze | -0.1742 | 0.2822 |
|  | Runny nose | 0.0350 | 0.8302 |
|  | Itchy eyes | -0.0842 | 0.6053 |
|  | Tearing | 0.1975 | 0.2218 |
|  | Red eyes | 0.2609 | 0.1039 |
|  | Eye pain | 0.0849 | 0.6024 |
|  | Cough | -0.0506 | 0.7564 |
|  | Breath holding | 0.1922 | 0.2347 |
|  | Wheezing | -0.0200 | 0.9027 |
|  | Pressure sensation | 0.0646 | 0.6920 |
| IL-10 | Total score | 0.1854 | 0.2521 |
|  | Nasal obstruction | 0.2603 | 0.1047 |
|  | Nasal pruritus | 0.1592 | 0.3266 |
|  | Sneeze | 0.0601 | 0.7125 |
|  | Runny nose | 0.2760 | 0.0847 |
|  | Itchy eyes | 0.1305 | 0.4222 |
|  | Tearing | 0.1296 | 0.4255 |
|  | Red eyes | 0.0024 | 0.9883 |
|  | Eye pain | 0.0167 | 0.9185 |
|  | Cough | 0.1525 | 0.3476 |
|  | Breath holding | -0.0211 | 0.8970 |
|  | Wheezing | 0.0597 | 0.7145 |
|  | Pressure sensation | -0.0746 | 0.6472 |
| IL-17 | Total score | -0.0357 | 0.8267 |
|  | Nasal obstruction | 0.1264 | 0.4372 |
|  | Nasal pruritus | 0.0337 | 0.8363 |
|  | Sneeze | -0.1212 | 0.4563 |
|  | Runny nose | -0.1304 | 0.4227 |
|  | Itchy eyes | -0.1273 | 0.4337 |
|  | Tearing | -0.0033 | 0.9839 |
|  | Red eyes | -0.0194 | 0.9053 |
|  | Eye pain | -0.0541 | 0.7404 |
|  | Cough | -0.0852 | 0.6010 |
|  | Breath holding | 0.3884 | **0.0133** |
|  | Wheezing | 0.2886 | 0.0709 |
|  | Pressure sensation | -0.1560 | 0.3365 |
| CCL2 | Total score | 0.0905 | 0.5787 |
|  | Nasal obstruction | 0.0778 | 0.6335 |
|  | Nasal pruritus | 0.1092 | 0.5025 |
|  | Sneeze | 0.0721 | 0.6585 |
|  | Runny nose | 0.0326 | 0.8415 |
|  | Itchy eyes | 0.0508 | 0.7555 |
|  | Tearing | 0.2219 | 0.1688 |
|  | Red eyes | 0.0963 | 0.5545 |
|  | Eye pain | -0.0761 | 0.6407 |
|  | Cough | -0.0178 | 0.9131 |
|  | Breath holding | -0.1061 | 0.5148 |
|  | Wheezing | 0.3922 | **0.0123** |
|  | Pressure sensation | -0.0353 | 0.8289 |
| CCL3 | Total score | 0.1715 | 0.2901 |
|  | Nasal obstruction | 0.2464 | 0.1253 |
|  | Nasal pruritus | 0.1238 | 0.4466 |
|  | Sneeze | 0.0319 | 0.8453 |
|  | Runny nose | 0.3457 | **0.0289** |
|  | Itchy eyes | 0.1585 | 0.3285 |
|  | Tearing | 0.0402 | 0.8053 |
|  | Red eyes | -0.0615 | 0.7063 |
|  | Eye pain | -0.1483 | 0.3610 |
|  | Cough | 0.3381 | **0.0329** |
|  | Breath holding | -0.0732 | 0.6537 |
|  | Wheezing | -0.0037 | 0.9820 |
|  | Pressure sensation | -0.0754 | 0.6438 |
| CCL4 | Total score | 0.1887 | 0.2436 |
|  | Nasal obstruction | 0.2603 | 0.1047 |
|  | Nasal pruritus | 0.1520 | 0.3491 |
|  | Sneeze | 0.0571 | 0.7264 |
|  | Runny nose | 0.3213 | **0.0432** |
|  | Itchy eyes | 0.1775 | 0.2732 |
|  | Tearing | 0.0747 | 0.6468 |
|  | Red eyes | -0.0170 | 0.9168 |
|  | Eye pain | -0.1314 | 0.4191 |
|  | Cough | 0.2880 | 0.0715 |
|  | Breath holding | -0.0630 | 0.6995 |
|  | Wheezing | 0.0784 | 0.6308 |
|  | Pressure sensation | -0.0694 | 0.6706 |
| CCL5 | Total score | 0.0981 | 0.5469 |
|  | Nasal obstruction | 0.2222 | 0.1683 |
|  | Nasal pruritus | 0.2019 | 0.2116 |
|  | Sneeze | -0.0197 | 0.9042 |
|  | Runny nose | -0.0837 | 0.6076 |
|  | Itchy eyes | -0.0530 | 0.7451 |
|  | Tearing | 0.2669 | 0.0959 |
|  | Red eyes | 0.1222 | 0.4524 |
|  | Eye pain | 0.1431 | 0.3782 |
|  | Cough | -0.0866 | 0.5952 |
|  | Breath holding | 0.2789 | 0.0814 |
|  | Wheezing | 0.2922 | 0.0673 |
|  | Pressure sensation | -0.1212 | 0.4564 |
| TNF-ɑ | Total score | 0.2765 | 0.0841 |
|  | Nasal obstruction | 0.4389 | **0.0046** |
|  | Nasal pruritus | 0.2578 | 0.1082 |
|  | Sneeze | -0.0030 | 0.9855 |
|  | Runny nose | 0.3006 | 0.0595 |
|  | Itchy eyes | 0.1118 | 0.4922 |
|  | Tearing | 0.3204 | **0.0438** |
|  | Red eyes | 0.1837 | 0.2566 |
|  | Eye pain | 0.0831 | 0.6102 |
|  | Cough | 0.2826 | 0.0773 |
|  | Breath holding | 0.1757 | 0.2781 |
|  | Wheezing | 0.1323 | 0.4157 |
|  | Pressure sensation | -0.1697 | 0.2952 |
| Tpo | Total score | 0.2012 | 0.2132 |
|  | Nasal obstruction | 0.3268 | **0.0395** |
|  | Nasal pruritus | 0.1972 | 0.2226 |
|  | Sneeze | -0.0349 | 0.8308 |
|  | Runny nose | 0.1387 | 0.3933 |
|  | Itchy eyes | 0.0145 | 0.9292 |
|  | Tearing | 0.3532 | **0.0254** |
|  | Red eyes | 0.2526 | 0.1158 |
|  | Eye pain | 0.2473 | 0.1240 |
|  | Cough | 0.0665 | 0.6833 |
|  | Breath holding | 0.2064 | 0.2013 |
|  | Wheezing | 0.1936 | 0.2312 |
|  | Pressure sensation | -0.1232 | 0.4488 |
| VEGF | Total score | 0.1176 | 0.4699 |
|  | Nasal obstruction | 0.2663 | 0.0967 |
|  | Nasal pruritus | 0.1129 | 0.4880 |
|  | Sneeze | -0.1131 | 0.4871 |
|  | Runny nose | 0.2076 | 0.1986 |
|  | Itchy eyes | -0.0287 | 0.8603 |
|  | Tearing | 0.1465 | 0.3671 |
|  | Red eyes | 0.0735 | 0.6520 |
|  | Eye pain | -0.0315 | 0.8469 |
|  | Cough | 0.2181 | 0.1764 |
|  | Breath holding | 0.1088 | 0.5039 |
|  | Wheezing | 0.2160 | 0.1807 |
|  | Pressure sensation | -0.1727 | 0.2866 |

The bold text indicates significance.

**Table S11** Correlation analysis of inflammatory cytokines in nasal secretions and serum

| Inflammatory cytokine in serum | Inflammatory cytokine in nasal secretions | r | *P* |
| --- | --- | --- | --- |
| CXCL5 | CXCL5 | -0.0531 | 0.7449 |
|  | FGF basic | 0.0387 | 0.8126 |
|  | G-CSF | 0.3274 | **0.0392** |
|  | GM-CSF | 0.0703 | 0.6664 |
|  | IFN-γ | 0.3336 | **0.0354** |
|  | IL-1β | 0.1551 | 0.3392 |
|  | IL-2 | 0.0911 | 0.5762 |
|  | IL-4 | 0.3209 | **0.0435** |
|  | IL-5 | 0.1160 | 0.4758 |
|  | IL-6 | 0.3182 | **0.0454** |
|  | CXCL8 | 0.0829 | 0.6111 |
|  | IL-10 | 0.1500 | 0.3554 |
|  | IL-17 | 0.2708 | 0.0910 |
|  | CCL2 | 0.2477 | 0.1234 |
|  | CCL3 | 0.1164 | 0.4746 |
|  | CCL4 | 0.1714 | 0.2903 |
|  | CCL5 | 0.1413 | 0.3845 |
|  | TNF-ɑ | 0.3079 | 0.0533 |
|  | Tpo | 0.2509 | 0.1183 |
|  | VEGF | 0.1956 | 0.2264 |
| FGF basic | CXCL5 | -0.1000 | 0.5392 |
|  | FGF basic | -0.0308 | 0.8501 |
|  | G-CSF | -0.1257 | 0.4395 |
|  | GM-CSF | -0.0904 | 0.5790 |
|  | IFN-γ | -0.1334 | 0.4119 |
|  | IL-1β | -0.1063 | 0.5139 |
|  | IL-2 | 0.0088 | 0.9569 |
|  | IL-4 | -0.1383 | 0.3948 |
|  | IL-5 | -0.1183 | 0.4671 |
|  | IL-6 | -0.0623 | 0.7025 |
|  | CXCL8 | -0.0594 | 0.7156 |
|  | IL-10 | -0.0713 | 0.6620 |
|  | IL-17 | -0.1451 | 0.3716 |
|  | CCL2 | -0.1548 | 0.3401 |
|  | CCL3 | -0.0823 | 0.6138 |
|  | CCL4 | -0.1277 | 0.4322 |
|  | CCL5 | 0.1056 | 0.5167 |
|  | TNF-ɑ | -0.1290 | 0.4276 |
|  | Tpo | -0.0784 | 0.6308 |
|  | VEGF | -0.0979 | 0.5480 |
| G-CSF | CXCL5 | -0.0934 | 0.5664 |
|  | FGF basic | -0.0490 | 0.7638 |
|  | G-CSF | 0.0978 | 0.5481 |
|  | GM-CSF | 0.0251 | 0.8778 |
|  | IFN-γ | 0.1253 | 0.4410 |
|  | IL-1β | 0.2654 | 0.0978 |
|  | IL-2 | -0.0635 | 0.6970 |
|  | IL-4 | 0.1120 | 0.4914 |
|  | IL-5 | -0.1275 | 0.4330 |
|  | IL-6 | 0.1446 | 0.3734 |
|  | CXCL8 | -0.0543 | 0.7391 |
|  | IL-10 | 0.0404 | 0.8046 |
|  | IL-17 | 0.1389 | 0.3926 |
|  | CCL2 | -0.1857 | 0.2514 |
|  | CCL3 | -0.0460 | 0.7778 |
|  | CCL4 | -0.0998 | 0.5401 |
|  | CCL5 | 0.2586 | 0.1071 |
|  | TNF-ɑ | 0.0794 | 0.6262 |
|  | Tpo | 0.2054 | 0.2035 |
|  | VEGF | 0.0539 | 0.7413 |
| GM-CSF | CXCL5 | -0.0186 | 0.9091 |
|  | FGF basic | -0.0122 | 0.9404 |
|  | G-CSF | 0.2421 | 0.1322 |
|  | GM-CSF | -0.0671 | 0.6809 |
|  | IFN-γ | 0.1962 | 0.2249 |
|  | IL-1β | 0.1953 | 0.2273 |
|  | IL-2 | 0.0020 | 0.9901 |
|  | IL-4 | 0.2157 | 0.1814 |
|  | IL-5 | -0.1635 | 0.3135 |
|  | IL-6 | 0.1111 | 0.4950 |
|  | CXCL8 | 0.2921 | 0.0675 |
|  | IL-10 | -0.0605 | 0.7109 |
|  | IL-17 | 0.2056 | 0.2032 |
|  | CCL2 | -0.0555 | 0.7337 |
|  | CCL3 | -0.0123 | 0.9399 |
|  | CCL4 | -0.0366 | 0.8228 |
|  | CCL5 | 0.2508 | 0.1185 |
|  | TNF-ɑ | 0.1585 | 0.3286 |
|  | Tpo | 0.1911 | 0.2376 |
|  | VEGF | 0.2323 | 0.1491 |
| IFN-γ | CXCL5 | -0.1608 | 0.3215 |
|  | FGF basic | -0.0634 | 0.6975 |
|  | G-CSF | -0.0943 | 0.5629 |
|  | GM-CSF | -0.1204 | 0.4591 |
|  | IFN-γ | -0.0557 | 0.7328 |
|  | IL-1β | 0.0895 | 0.5830 |
|  | IL-2 | 0.0527 | 0.7466 |
|  | IL-4 | -0.0666 | 0.6830 |
|  | IL-5 | 0.0631 | 0.6990 |
|  | IL-6 | -0.0200 | 0.9026 |
|  | CXCL8 | -0.2048 | 0.2050 |
|  | IL-10 | 0.0080 | 0.9608 |
|  | IL-17 | -0.0080 | 0.9611 |
|  | CCL2 | -0.0201 | 0.9019 |
|  | CCL3 | -0.0405 | 0.8042 |
|  | CCL4 | -0.0600 | 0.7132 |
|  | CCL5 | 0.0294 | 0.8573 |
|  | TNF-ɑ | -0.0793 | 0.6267 |
|  | Tpo | 0.0137 | 0.9329 |
|  | VEGF | 0.0247 | 0.8800 |
| IL-1β | CXCL5 | -0.0841 | 0.6060 |
|  | FGF basic | 0.0873 | 0.5923 |
|  | G-CSF | -0.0015 | 0.9926 |
|  | GM-CSF | -0.0897 | 0.5819 |
|  | IFN-γ | -0.0387 | 0.8127 |
|  | IL-1β | -0.0656 | 0.6874 |
|  | IL-2 | 0.1366 | 0.4005 |
|  | IL-4 | -0.0451 | 0.7821 |
|  | IL-5 | -0.0401 | 0.8062 |
|  | IL-6 | 0.0255 | 0.8759 |
|  | CXCL8 | 0.0504 | 0.7574 |
|  | IL-10 | -0.0910 | 0.5767 |
|  | IL-17 | 0.0048 | 0.9766 |
|  | CCL2 | -0.0460 | 0.7779 |
|  | CCL3 | -0.0818 | 0.6156 |
|  | CCL4 | -0.0925 | 0.5703 |
|  | CCL5 | 0.2031 | 0.2088 |
|  | TNF-ɑ | -0.0246 | 0.8804 |
|  | Tpo | -0.1109 | 0.4958 |
|  | VEGF | -0.0516 | 0.7518 |
| IL-2 | CXCL5 | -0.0530 | 0.7454 |
|  | FGF basic | -0.2056 | 0.2032 |
|  | G-CSF | 0.2742 | 0.0869 |
|  | GM-CSF | 0.3708 | **0.0185** |
|  | IFN-γ | 0.2714 | 0.0903 |
|  | IL-1β | 0.0728 | 0.6553 |
|  | IL-2 | 0.1442 | 0.3748 |
|  | IL-4 | 0.2538 | 0.1140 |
|  | IL-5 | -0.1642 | 0.3114 |
|  | IL-6 | 0.2255 | 0.1617 |
|  | CXCL8 | 0.2085 | 0.1968 |
|  | IL-10 | 0.0847 | 0.6032 |
|  | IL-17 | 0.0338 | 0.8362 |
|  | CCL2 | 0.0624 | 0.7020 |
|  | CCL3 | 0.0868 | 0.5944 |
|  | CCL4 | 0.0734 | 0.6526 |
|  | CCL5 | 0.1533 | 0.3449 |
|  | TNF-ɑ | 0.2043 | 0.2060 |
|  | Tpo | 0.2970 | 0.0628 |
|  | VEGF | 0.2256 | 0.1616 |
| IL-4 | CXCL5 | 0.1046 | 0.5205 |
|  | FGF basic | -0.0365 | 0.8233 |
|  | G-CSF | 0.2297 | 0.1539 |
|  | GM-CSF | 0.1260 | 0.4386 |
|  | IFN-γ | 0.2126 | 0.1878 |
|  | IL-1β | -0.0054 | 0.9735 |
|  | IL-2 | 0.2262 | 0.1605 |
|  | IL-4 | 0.2223 | 0.1680 |
|  | IL-5 | -0.0967 | 0.5529 |
|  | IL-6 | 0.1514 | 0.3510 |
|  | CXCL8 | 0.2275 | 0.1580 |
|  | IL-10 | 0.1779 | 0.2720 |
|  | IL-17 | -0.0081 | 0.9604 |
|  | CCL2 | -0.0289 | 0.8596 |
|  | CCL3 | 0.0906 | 0.5782 |
|  | CCL4 | 0.0724 | 0.6572 |
|  | CCL5 | 0.1751 | 0.2799 |
|  | TNF-ɑ | 0.2217 | 0.1692 |
|  | Tpo | 0.3216 | **0.0430** |
|  | VEGF | 0.0872 | 0.5924 |
| IL-5 | CXCL5 | -0.3772 | **0.0164** |
|  | FGF basic | 0.0500 | 0.7592 |
|  | G-CSF | -0.0120 | 0.9417 |
|  | GM-CSF | 0.2008 | 0.2140 |
|  | IFN-γ | -0.0731 | 0.6539 |
|  | IL-1β | -0.0027 | 0.9870 |
|  | IL-2 | -0.1758 | 0.2779 |
|  | IL-4 | -0.0194 | 0.9057 |
|  | IL-5 | -0.0939 | 0.5644 |
|  | IL-6 | 0.1385 | 0.3941 |
|  | CXCL8 | -0.0217 | 0.8941 |
|  | IL-10 | 0.1200 | 0.4609 |
|  | IL-17 | 0.1275 | 0.4329 |
|  | CCL2 | -0.0068 | 0.9666 |
|  | CCL3 | 0.1763 | 0.2765 |
|  | CCL4 | 0.1596 | 0.3252 |
|  | CCL5 | 0.0354 | 0.8284 |
|  | TNF-ɑ | 0.0138 | 0.9324 |
|  | Tpo | -0.0197 | 0.9040 |
|  | VEGF | 0.1729 | 0.2860 |
| IL-6 | CXCL5 | -0.1320 | 0.4167 |
|  | FGF basic | 0.0108 | 0.9470 |
|  | G-CSF | -0.0482 | 0.7676 |
|  | GM-CSF | -0.0482 | 0.7678 |
|  | IFN-γ | -0.0702 | 0.6670 |
|  | IL-1β | -0.0912 | 0.5757 |
|  | IL-2 | 0.1598 | 0.3247 |
|  | IL-4 | -0.0611 | 0.7082 |
|  | IL-5 | -0.0619 | 0.7045 |
|  | IL-6 | -0.0171 | 0.9167 |
|  | CXCL8 | -0.0375 | 0.8183 |
|  | IL-10 | 0.0452 | 0.7816 |
|  | IL-17 | -0.0695 | 0.6698 |
|  | CCL2 | -0.1271 | 0.4343 |
|  | CCL3 | -0.0078 | 0.9620 |
|  | CCL4 | -0.0468 | 0.7741 |
|  | CCL5 | 0.1048 | 0.5199 |
|  | TNF-ɑ | -0.0437 | 0.7887 |
|  | Tpo | 0.0097 | 0.9526 |
|  | VEGF | -0.0329 | 0.8401 |
| CXCL8 | CXCL5 | -0.1710 | 0.2915 |
|  | FGF basic | -0.0177 | 0.9137 |
|  | G-CSF | 0.0466 | 0.7752 |
|  | GM-CSF | -0.0500 | 0.7593 |
|  | IFN-γ | -0.0101 | 0.9505 |
|  | IL-1β | -0.0300 | 0.8544 |
|  | IL-2 | 0.1301 | 0.4236 |
|  | IL-4 | -0.0081 | 0.9606 |
|  | IL-5 | -0.0950 | 0.5600 |
|  | IL-6 | 0.0719 | 0.6593 |
|  | CXCL8 | 0.0010 | 0.9953 |
|  | IL-10 | -0.0040 | 0.9804 |
|  | IL-17 | 0.0292 | 0.8578 |
|  | CCL2 | -0.1442 | 0.3746 |
|  | CCL3 | 0.0287 | 0.8603 |
|  | CCL4 | -0.0183 | 0.9107 |
|  | CCL5 | 0.1900 | 0.2402 |
|  | TNF-ɑ | 0.0218 | 0.8937 |
|  | Tpo | -0.0592 | 0.7166 |
|  | VEGF | 0.0380 | 0.8158 |
| IL-10 | CXCL5 | -0.1522 | 0.3486 |
|  | FGF basic | -0.0591 | 0.7174 |
|  | G-CSF | -0.1811 | 0.2635 |
|  | GM-CSF | -0.1507 | 0.3532 |
|  | IFN-γ | -0.1630 | 0.3148 |
|  | IL-1β | -0.1273 | 0.4336 |
|  | IL-2 | 0.0106 | 0.9485 |
|  | IL-4 | -0.1673 | 0.3022 |
|  | IL-5 | 0.1875 | 0.2465 |
|  | IL-6 | -0.1609 | 0.3213 |
|  | CXCL8 | -0.1888 | 0.2433 |
|  | IL-10 | -0.1188 | 0.4654 |
|  | IL-17 | -0.2034 | 0.2080 |
|  | CCL2 | -0.1813 | 0.2629 |
|  | CCL3 | -0.0243 | 0.8818 |
|  | CCL4 | -0.0636 | 0.6968 |
|  | CCL5 | -0.1177 | 0.4693 |
|  | TNF-ɑ | -0.1561 | 0.3362 |
|  | Tpo | 0.0499 | 0.7597 |
|  | VEGF | -0.0713 | 0.6620 |
| IL-17 | CXCL5 | 0.1148 | 0.4805 |
|  | FGF basic | -0.0099 | 0.9519 |
|  | G-CSF | 0.0802 | 0.6229 |
|  | GM-CSF | 0.1431 | 0.3783 |
|  | IFN-γ | 0.1555 | 0.3380 |
|  | IL-1β | 0.0169 | 0.9178 |
|  | IL-2 | -0.0125 | 0.9389 |
|  | IL-4 | 0.1322 | 0.4161 |
|  | IL-5 | -0.1610 | 0.3209 |
|  | IL-6 | 0.0170 | 0.9172 |
|  | CXCL8 | 0.1648 | 0.3094 |
|  | IL-10 | 0.0268 | 0.8696 |
|  | IL-17 | -0.1853 | 0.2524 |
|  | CCL2 | 0.1682 | 0.2994 |
|  | CCL3 | 0.0004 | 0.9980 |
|  | CCL4 | -0.0015 | 0.9928 |
|  | CCL5 | 0.0406 | 0.8037 |
|  | TNF-ɑ | 0.1065 | 0.5131 |
|  | Tpo | 0.2167 | 0.1792 |
|  | VEGF | 0.0553 | 0.7347 |
| CCL2 | CXCL5 | -0.0174 | 0.9152 |
|  | FGF basic | -0.1811 | 0.2635 |
|  | G-CSF | 0.2267 | 0.1595 |
|  | GM-CSF | 0.2815 | 0.0785 |
|  | IFN-γ | 0.1905 | 0.2389 |
|  | IL-1β | -0.1159 | 0.4763 |
|  | IL-2 | 0.2081 | 0.1976 |
|  | IL-4 | 0.1895 | 0.2414 |
|  | IL-5 | -0.1465 | 0.3670 |
|  | IL-6 | 0.1653 | 0.3082 |
|  | CXCL8 | 0.2877 | 0.0719 |
|  | IL-10 | 0.2475 | 0.1236 |
|  | IL-17 | -0.0263 | 0.8719 |
|  | CCL2 | 0.0386 | 0.8131 |
|  | CCL3 | 0.1601 | 0.3238 |
|  | CCL4 | 0.1431 | 0.3785 |
|  | CCL5 | 0.1058 | 0.5157 |
|  | TNF-ɑ | 0.1725 | 0.2871 |
|  | Tpo | 0.1751 | 0.2800 |
|  | VEGF | 0.1748 | 0.2807 |
| CCL3 | CXCL5 | -0.0684 | 0.6749 |
|  | FGF basic | 0.0622 | 0.7028 |
|  | G-CSF | 0.0312 | 0.8486 |
|  | GM-CSF | -0.0478 | 0.7698 |
|  | IFN-γ | -0.0235 | 0.8856 |
|  | IL-1β | -0.0587 | 0.7192 |
|  | IL-2 | 0.1041 | 0.5228 |
|  | IL-4 | -0.0219 | 0.8932 |
|  | IL-5 | -0.1360 | 0.4026 |
|  | IL-6 | 0.0897 | 0.5822 |
|  | CXCL8 | 0.0837 | 0.6078 |
|  | IL-10 | -0.0259 | 0.8741 |
|  | IL-17 | 0.0473 | 0.7719 |
|  | CCL2 | -0.1412 | 0.3848 |
|  | CCL3 | -0.0124 | 0.9395 |
|  | CCL4 | -0.0477 | 0.7701 |
|  | CCL5 | 0.2709 | 0.0908 |
|  | TNF-ɑ | -0.0002 | 0.9992 |
|  | Tpo | -0.1027 | 0.5282 |
|  | VEGF | -0.0264 | 0.8717 |
| CCL4 | CXCL5 | 0.1193 | 0.4636 |
|  | FGF basic | 0.0259 | 0.8740 |
|  | G-CSF | 0.3046 | 0.0560 |
|  | GM-CSF | -0.0028 | 0.9862 |
|  | IFN-γ | 0.1982 | 0.2203 |
|  | IL-1β | -0.0806 | 0.6210 |
|  | IL-2 | 0.1954 | 0.2269 |
|  | IL-4 | 0.2339 | 0.1463 |
|  | IL-5 | -0.2114 | 0.1905 |
|  | IL-6 | 0.3342 | **0.0351** |
|  | CXCL8 | 0.1634 | 0.3138 |
|  | IL-10 | 0.2043 | 0.2060 |
|  | IL-17 | 0.1093 | 0.5019 |
|  | CCL2 | -0.0069 | 0.9664 |
|  | CCL3 | 0.1991 | 0.2181 |
|  | CCL4 | 0.1680 | 0.3002 |
|  | CCL5 | 0.2564 | 0.1102 |
|  | TNF-ɑ | 0.2594 | 0.1061 |
|  | Tpo | 0.1716 | 0.2898 |
|  | VEGF | 0.1776 | 0.2728 |
| CCL5 | CXCL5 | 0.1857 | 0.2514 |
|  | FGF basic | -0.0834 | 0.6090 |
|  | G-CSF | 0.0133 | 0.9351 |
|  | GM-CSF | 0.1949 | 0.2281 |
|  | IFN-γ | -0.0478 | 0.7697 |
|  | IL-1β | -0.2211 | 0.1703 |
|  | IL-2 | 0.1401 | 0.3884 |
|  | IL-4 | -0.0479 | 0.7693 |
|  | IL-5 | -0.1851 | 0.2529 |
|  | IL-6 | -0.0685 | 0.6743 |
|  | CXCL8 | 0.1266 | 0.4364 |
|  | IL-10 | 0.1295 | 0.4257 |
|  | IL-17 | -0.1685 | 0.2987 |
|  | CCL2 | -0.0570 | 0.7266 |
|  | CCL3 | -0.0325 | 0.8422 |
|  | CCL4 | -0.0653 | 0.6891 |
|  | CCL5 | -0.0600 | 0.7131 |
|  | TNF-ɑ | -0.0591 | 0.7171 |
|  | Tpo | -0.1192 | 0.4639 |
|  | VEGF | -0.0852 | 0.6011 |
| TNF-ɑ | CXCL5 | -0.1278 | 0.4319 |
|  | FGF basic | -0.0254 | 0.8765 |
|  | G-CSF | -0.0614 | 0.7068 |
|  | GM-CSF | -0.2176 | 0.1774 |
|  | IFN-γ | -0.1411 | 0.3853 |
|  | IL-1β | -0.1386 | 0.3936 |
|  | IL-2 | -0.1342 | 0.4090 |
|  | IL-4 | -0.1289 | 0.4279 |
|  | IL-5 | -0.2490 | 0.1213 |
|  | IL-6 | -0.0756 | 0.6428 |
|  | CXCL8 | 0.1117 | 0.4926 |
|  | IL-10 | -0.2627 | 0.1015 |
|  | IL-17 | -0.0814 | 0.6174 |
|  | CCL2 | -0.1878 | 0.2458 |
|  | CCL3 | -0.2885 | 0.0710 |
|  | CCL4 | -0.3350 | **0.0346** |
|  | CCL5 | 0.1518 | 0.3498 |
|  | TNF-ɑ | -0.1941 | 0.2302 |
|  | Tpo | -0.0222 | 0.8918 |
|  | VEGF | -0.1466 | 0.3666 |
| Tpo | CXCL5 | 0.3327 | **0.0360** |
|  | FGF basic | -0.0551 | 0.7358 |
|  | G-CSF | 0.1681 | 0.2999 |
|  | GM-CSF | -0.0458 | 0.7788 |
|  | IFN-γ | 0.1475 | 0.3636 |
|  | IL-1β | -0.0902 | 0.5801 |
|  | IL-2 | 0.0467 | 0.7750 |
|  | IL-4 | 0.1389 | 0.3926 |
|  | IL-5 | -0.1515 | 0.3506 |
|  | IL-6 | 0.0681 | 0.6763 |
|  | CXCL8 | 0.2401 | 0.1356 |
|  | IL-10 | 0.1374 | 0.3979 |
|  | IL-17 | 0.0104 | 0.9491 |
|  | CCL2 | 0.0700 | 0.6677 |
|  | CCL3 | 0.0293 | 0.8574 |
|  | CCL4 | 0.0429 | 0.7927 |
|  | CCL5 | 0.1246 | 0.4437 |
|  | TNF-ɑ | 0.1213 | 0.4559 |
|  | Tpo | 0.1455 | 0.3704 |
|  | VEGF | -0.0746 | 0.6475 |
| VEGF | CXCL5 | 0.1600 | 0.3239 |
|  | FGF basic | -0.0586 | 0.7195 |
|  | G-CSF | -0.0884 | 0.5874 |
|  | GM-CSF | 0.0913 | 0.5754 |
|  | IFN-γ | -0.1164 | 0.4746 |
|  | IL-1β | -0.2137 | 0.1854 |
|  | IL-2 | -0.1917 | 0.2361 |
|  | IL-4 | -0.1112 | 0.4946 |
|  | IL-5 | -0.1824 | 0.2599 |
|  | IL-6 | -0.2133 | 0.1864 |
|  | CXCL8 | 0.1433 | 0.3777 |
|  | IL-10 | -0.1296 | 0.4253 |
|  | IL-17 | -0.1472 | 0.3648 |
|  | CCL2 | -0.1320 | 0.4168 |
|  | CCL3 | -0.1318 | 0.4176 |
|  | CCL4 | -0.1700 | 0.2942 |
|  | CCL5 | -0.0609 | 0.7088 |
|  | TNF-ɑ | -0.1230 | 0.4495 |
|  | Tpo | 0.0338 | 0.8359 |
|  | VEGF | -0.1519 | 0.3493 |

The bold text indicates significance.
